# Immuno-Modulatory Effects of Intervertebral Disc Cells

**DOI:** 10.3389/fcell.2022.924692

**Published:** 2022-06-29

**Authors:** Paola Bermudez-Lekerika, Katherine B. Crump, Sofia Tseranidou, Andrea Nüesch, Exarchos Kanelis, Ahmad Alminnawi, Laura Baumgartner, Estefano Muñoz-Moya, Roger Compte, Francesco Gualdi, Leonidas G. Alexopoulos, Liesbet Geris, Karin Wuertz-Kozak, Christine L. Le Maitre, Jérôme Noailly, Benjamin Gantenbein

**Affiliations:** ^1^ Tissue Engineering for Orthopaedics and Mechanobiology, Bone and Joint Program, Department for BioMedical Research (DBMR), Faculty of Medicine, University of Bern, Bern, Switzerland; ^2^ Department of Orthopaedic Surgery and Traumatology, Inselspital, Bern University Hospital, Medical Faculty, University of Bern, Bern, Switzerland; ^3^ BCN MedTech, Universitat Pompeu Fabra, Barcelona, Spain; ^4^ Biomolecular Sciences Research Centre, Sheffield Hallam University, Sheffield, United Kingdom; ^5^ ProtATonce Ltd., Athens, Greece; ^6^ School of Mechanical Engineering, National Technical University of Athens, Zografou, Greece; ^7^ GIGA In Silico Medicine, University of Liège, Liège, Belgium; ^8^ Skeletal Biology and Engineering Research Center, KU Leuven, Leuven, Belgium; ^9^ Twin Research and Genetic Epidemiology, St Thomas’ Hospital, King’s College London, London, United Kingdom; ^10^ Institut Hospital Del Mar D’Investigacions Mèdiques (IMIM), Barcelona, Spain; ^11^ Biomechanics Research Unit, KU Leuven, Leuven, Belgium; ^12^ Department of Biomedical Engineering, Rochester Institute of Technology, Rochester, NY, United States; ^13^ Spine Center, Schön Klinik München Harlaching Academic Teaching Hospital and Spine Research Institute of the Paracelsus Private Medical University Salzburg (Austria), Munich, Germany

**Keywords:** intervertebral disc degeneration, low back pain, inflammation, catabolism, immune-privileged microenvironment, GWAS, artificial intelligence–AI, agent-based model (ABM)

## Abstract

Low back pain is a highly prevalent, chronic, and costly medical condition predominantly triggered by intervertebral disc degeneration (IDD). IDD is often caused by structural and biochemical changes in intervertebral discs (IVD) that prompt a pathologic shift from an anabolic to catabolic state, affecting extracellular matrix (ECM) production, enzyme generation, cytokine and chemokine production, neurotrophic and angiogenic factor production. The IVD is an immune-privileged organ. However, during degeneration immune cells and inflammatory factors can infiltrate through defects in the cartilage endplate and annulus fibrosus fissures, further accelerating the catabolic environment. Remarkably, though, catabolic ECM disruption also occurs in the absence of immune cell infiltration, largely due to native disc cell production of catabolic enzymes and cytokines. An unbalanced metabolism could be induced by many different factors, including a harsh microenvironment, biomechanical cues, genetics, and infection. The complex, multifactorial nature of IDD brings the challenge of identifying key factors which initiate the degenerative cascade, eventually leading to back pain. These factors are often investigated through methods including animal models, 3D cell culture, bioreactors, and computational models. However, the crosstalk between the IVD, immune system, and shifted metabolism is frequently misconstrued, often with the assumption that the presence of cytokines and chemokines is synonymous to inflammation or an immune response, which is not true for the intact disc. Therefore, this review will tackle immunomodulatory and IVD cell roles in IDD, clarifying the differences between cellular involvements and implications for therapeutic development and assessing models used to explore inflammatory or catabolic IVD environments.

## Introduction

### Epidemiology of Intervertebral Disc Degeneration

Low back pain (LBP) is the largest cause of morbidity worldwide, affecting approximately 80% of people from Western countries during their lifetime and resulting in 5 million disability-adjusted life-years in young adults (GBD 2017 Disease and Injury Incidence and Prevalence Collaborators, 2018). Lower intervertebral disc degeneration (IDD) is the cause of around half of all LBP cases in young adults; however not all cases of IDD result in LBP (Baumgartner et al., 2021c). Although IDD prevalence increases progressively with age, IDD is common in subjects younger than 30 years old, conveying those various other factors besides age, such as excessive or uneven mechanical load, obesity, genetics, nutrition, trauma, and gender are involved (Hoogendoorn et al., 2000; Paassilta et al., 2001; Pincus et al., 2002; Cheung et al., 2009; Samartzis et al., 2012; Teraguchi et al., 2014; Parenteau et al., 2021). For example, studies have shown that women experience greater pain and disability than men when they are treated for IDD (MacLean et al., 2020). Additionally, LBP prevalence in females after menopause further increases in comparison to men at comparable ages (Wáng et al., 2016). Further, it is unclear whether occupation-related heavy physical loading is an important risk factor for IDD, as studies have contradictory conclusions (Bongers et al., 1990; Videman and Battié, 1999). Some studies have found IDD is significantly more common in athletes compared to the general population (Swärd et al., 1991). However, various twin studies that have been conducted suggest that occupation or sport related risk factors have only a minor role in IDD, while genetic influences were found to play a greater role in predicting degeneration (Battié et al., 2004). On the other hand, obesity is associated with increased IDD severity and extent, likely due to altered biomechanical and/or biological processes such as those driven by adipokines (Samartzis et al., 2012; Li W. et al., 2022). Due to the complexity and multifactorial nature of IDD, the initiating and risk factors are poorly understood, which critically hampers proper LBP patient stratification and limits the development of personalized therapies.

### The Structure of the Intervertebral Disc

**FIGURE 1:**
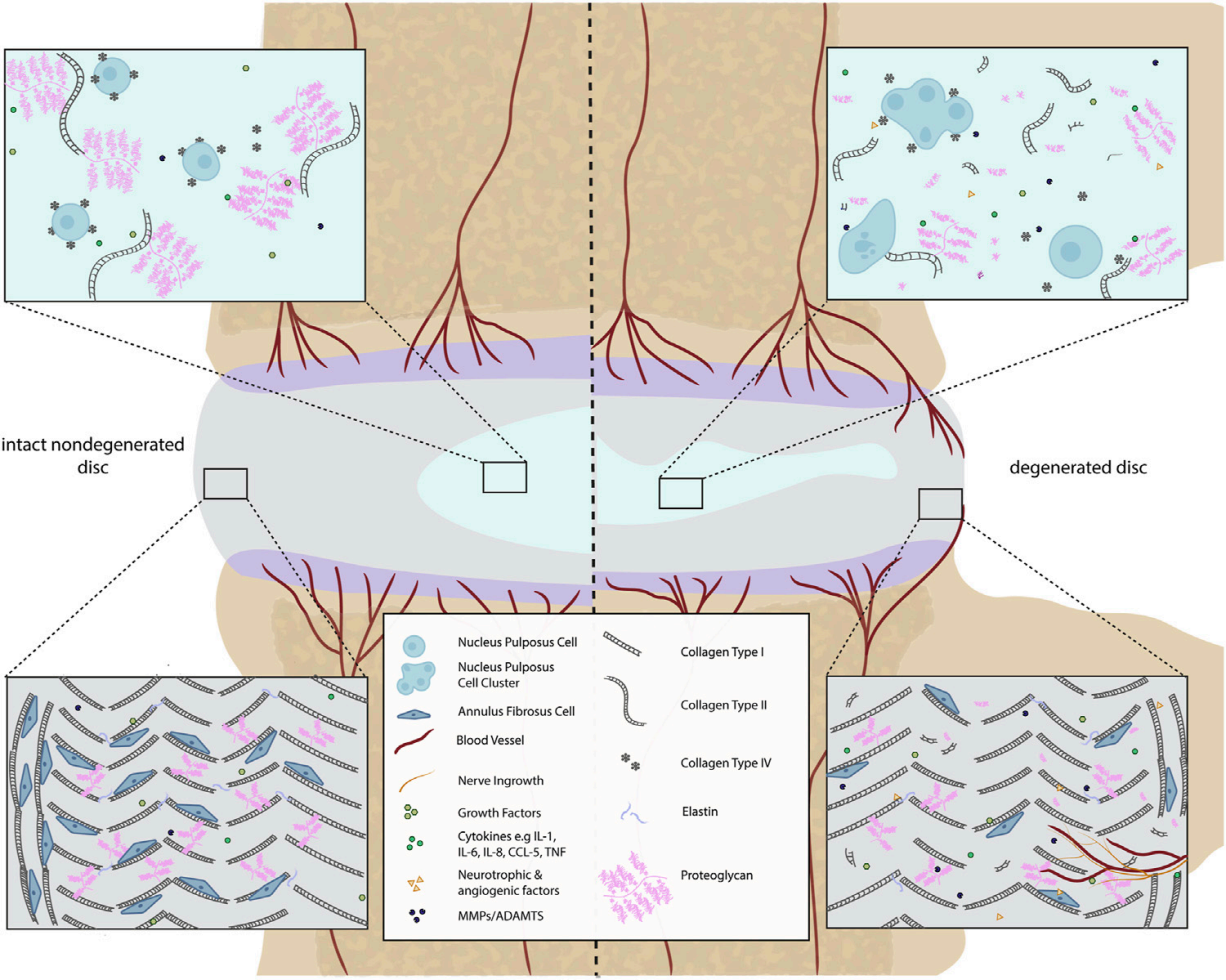
Comparison of a healthy and a degenerated IVD disc (focused on ECM components). In the intact IVD, the NP matrix mostly contains proteoglycans (PG) and non-oriented collagen type II fibers. The proteoglycans contain negatively charged sulfated groups leading to an intradiscal osmotic pressure crucial for the basal hydration of the NP and the biomechanical function of the IVD. Within the degenerated disc, the total content of PG decreases. Small non-aggregating PGs are present. This drop-in PG content negatively affects the swelling capacity of the disc. Additionally, during disc degeneration, the production of catabolic cytokines, matrix-degrading enzymes, and neurotrophic as well as angiogenic factors occur due to cellular changes. This can lead to blood and nerve vessel ingrowth in the AF. The AF is composed of highly oriented concentric lamella of type I collagen whereas the cell density is higher in intact than in degenerated discs.

The intervertebral disc (IVD) is the largest avascular organ in the human body with blood vessels only present in the outer annulus fibrosus (AF) and boney end plates, with all nutrient and waste exchange taking place via diffusion through the dense extracellular matrix of the disc (Urban 2002). Located between the vertebrae within the spine, the IVD consists of three highly hydrated, major tissues: 1) the nucleus pulposus (NP), 2) the annulus fibrosus (AF), and 3) the cartilage endplate (CEP). The central and proteoglycan-rich NP lies between the cranial and caudal CEPs and is laterally constrained by the peripheral and fiber-reinforced AF (Figure 1). This specialized composition and structure of the IVD ensures both trunk movements and resistance to high mechanical loads. The normal human IVD contains nucleus pulposus cells and annulus fibrosus cells within the NP and AF, respectively, with AF cells becoming more elongated and fibroblast-like towards the periphery. Cells occupy 1% volume of the disc, though are crucial in maintaining the balance between anabolic activity such as the production of proteoglycans and collagens type I and II, and the procatabolic effects of factors involved in ECM turnover, including metalloproteinases, prostaglandins, and nitric oxide (Kang et al., 1997). Furthermore, mechanical loads are thought to influence ECM homeostasis, where both excessive and insufficient loads lead to catabolism (Vergroesen et al., 2015). Due to the avascularity of the IVD, the environment is hypoxic, where the oxygen tension in the IVD is considered between 1 and 5% (Yao et al., 2016; Yao et al., 2017).

In comparison to NP and AF tissues, the CEP receives far less attention in the literature; however, it is a vital tissue when discussing LBP. Lakstins et al. (2021) demonstrated that imperfections and weakness in the CEP can be a better anticipator of pain than IVD degeneration because chemical changes to the CEP are directly related to intervertebral disc degeneration (IDD) (Yao et al., 2016). The CEP is rich in collagen type II (Yao et al., 2016) and performs both mechanical and chemical functions (Roberts et al., 1989; Lakstins et al., 2021). Mechanically, the CEP acts as a physical filter preventing macromolecules from escaping the disc through the subchondral bone and is considered important in controlling the hydration of the disc under mechanical loads (Roberts et al., 1989; Moore 2000; Ruiz Wills et al., 2018). Chemically, the CEP allows metabolites, small molecules and waste to travel between the IVD and neighboring blood vessels in the bony endplates (Roberts et al., 1989; Turgut et al., 2003; Yao et al., 2016; Ruiz Wills et al., 2018; Zuo et al., 2019; Sun et al., 2020; Lakstins et al., 2021).

The diffusivity of solutes through the CEP and towards the IVD depends greatly on their size and ionic charge. The healthy IVD is negatively charged due to the high proteoglycan concentration (Moore 2000; Pfannkuche et al., 2020). Therefore, only small, neutrally charged solutes such as glucose and oxygen, as well as cations such as sodium and calcium can penetrate the disc, but small anions such as sulphate and chloride ions can only cross through the CEP. In turn, large, neutrally charged solutes such as antibodies and enzymes usually cannot penetrate the healthy IVD (Moore 2000).

### Intervertebral Disc Degeneration

Regarding disc morphology, as the IVD degenerates, it becomes more difficult to distinguish the boundaries between the AF and the NP. This loss of a distinct boundary worsens with age, as the nucleus loses its gel-like quality and becomes more fibrotic (Buckwalter 1995) which was seen as a common degenerative feature across all species (Dahia et al., 2021). Another significant biochemical change during disc degeneration is the loss of proteoglycans, which are necessary to provide the osmotic resistance for the IVD to withstand compressive loads and keep the disc hydrated (Knudson and Knudson, 2001). Such significant changes (loss of water content (Lyons et al., 1981) and disc height (Frobin et al., 2001)) in disc behavior strongly influence other spinal structures and may negatively impact their function and predispose them to injury.

During IDD, the CEP becomes thinner and fissured, with lower collagen and glycosaminoglycan (GAG) content (Turgut et al., 2003; Sun et al., 2020). This change in morphology affects the physiology and the performance of the CEP (Hamilton et al., 2006; Roberts et al., 2006) altering its permeability (Roberts et al., 2006; Yao et al., 2016). Furthermore, the CEP can lose its connection to the vasculature (Moon et al., 2013), which immunohistochemistry has shown leads to blood vessel and nerve fiber infiltration into the IVD through the CEP and subchondral bone and through fissures in the AF (Freemont et al., 1997; Roberts et al., 2006; Binch et al., 2015b; Yao et al., 2016; Lama et al., 2018; Sun et al., 2020). Moreover, the crosstalk between IVD and the bone marrow is facilitated due to the CEP damage (Dudli et al., 2016), causing possible adjacent “Modic discs”. Modic changes (MC) are defined as magnetic resonance imaging (MRI) signal alterations in the vertebral bone marrow close to a degenerated disc. There are several different types of MC, with MC1 fibrotic lesion having the highest association with pain, followed by MC2. MC3 are rare and often asymptomatic. MC1 and MC2 are commonly accompanied by persistent inflammatory stimulus. In addition, MC related pain could be related to the neovascularization and neurogenesis due to the increase in growth factor expression by blood vessels and disc cells and inflammatory cytokines (Rätsep et al., 2013; Sun et al., 2013; Dudli et al., 2018) which lead to increased expression of neurotrophic factors (Freemont et al., 2002; Purmessur et al., 2008; Binch et al., 2014) (Figure 1).

During disc degeneration, the balance between anabolism and catabolism is dysregulated, showing decreased synthesis of normal matrix, of collagen type II and aggrecan and increased presence of matrix degrading enzymes, reviewed previously by Baumgartner et al. (2021c). Moreover, several studies have reported decreased NP cell proliferation under catabolic cytokine stimulation (Wang et al., 2013; Li et al., 2019; Lin and Lin, 2020). Similarly, during the shift from anabolic to catabolic cell activity in the disc, the presence of these cytokines is also related to NP and AF cell apoptosis, (Hu et al., 2017; Yu et al., 2018; Zhang J. et al., 2019; Zhang S. et al., 2019).

These changes have been shown, at least in part, to be modulated by pro-catabolic cytokines in numerous studies, which are often referred to as inflammatory features, in the literature. However, since these factors are produced by native disc cells (NP, AF and CEP) in intact discs, this catabolic response can be easily misconstrued as an inflammatory response. Therefore, the aim of this review is to tackle immunomodulatory and IVD cell roles in IDD and clarifying the differences between cellular involvements. Furthermore, different *in-silico, in-vivo* and *in-vitro* models used to explore inflammatory or catabolic IVD environments will be discussed.

## Cross-Talk Between the Immune System and IVD in IDD

As mentioned, the IVD is the largest avascular organ with blood vessels only present in the outer AF and bony end plates, so all metabolite exchange takes place via diffusion through the dense extracellular matrix (ECM) of the IVD. The dense ECM of the IVD inhibits blood vessel ingrowth both mechanically by having a high physical pressure, and chemically through high proteoglycan concentration (Johnson et al., 2002; Johnson et al., 2005), which combined with secretory inhibitors prevent nerve and blood vessel ingrowth in non-degenerate discs (Tolofari et al., 2010; Binch et al., 2015a). The AF and the CEP, along with the secretory inhibitors of angiogenesis, are defined as the blood-NP barrier (BNB), which strongly isolates the NP from the circulation and thus the host immune system (Sun et al., 2020).

**FIGURE 2:**
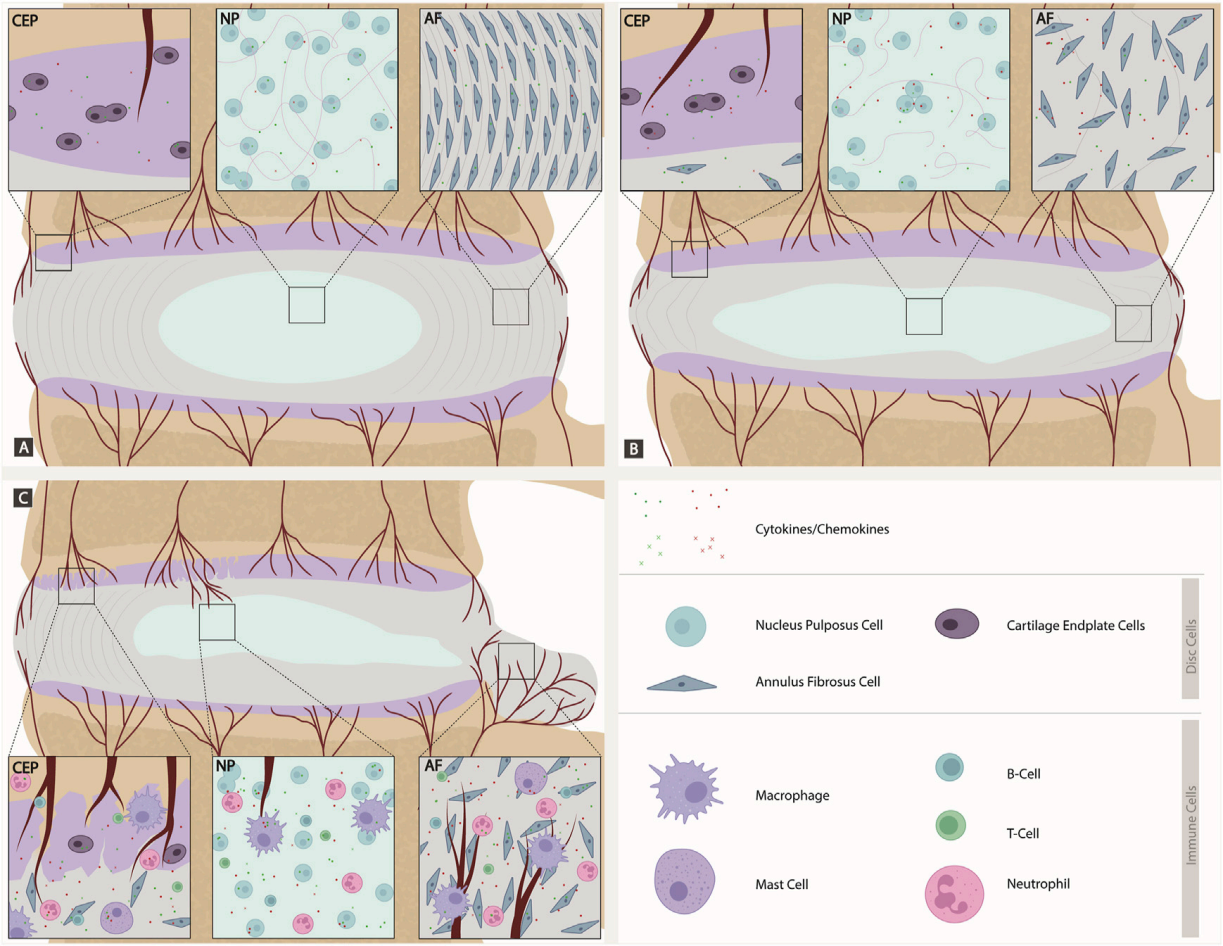
Comparison of a healthy, degenerated, and herniated IVD discs (focused on cellular involvement). **(A)** Intact IVD: Native disc cells produce a plethora of cytokines and chemokines expressing the corresponding receptors and maintaining homeostasis in a para and autocrine manner. The CEP is intact with blood vessels. The NP has a high number of proteoglycans. AF cells aligned. **(B)** Degenerated IVD: Shift to catabolic environment. Cytokines are expressed by disc cells themselves. The CEP has a higher amount of blood vessels than in the intact IVD. Proteoglycan number decreases in the NP. In the AF, there is a loss of alignment and support for AF cells. **(C)** Herniated IVD with crack in CEP: As soon as the AF or CEP is ruptured during injury or disc degeneration, a route for migration of immune cells into the IVD is provided. Immune cells, including T cells, B cells, macrophages, neutrophils and mast cells, contribute to an inflamed environment within the disc, further increasing the cytokine and chemokine expression and leading to a viscous circle of inflammatory driven catabolism. A crack in CEP allows blood vessels to grow into the AF and NP. The AF herniates/bulges, which is where blood vessel in-growth primarily occurs.

Where both AF and CEP are intact, the IVD has been described as an immuno-privileged tissue (Sun et al., 2020) with a lack of immune cells (Figure 2). However, this is often confused as the native cells of the disc (i.e., the NP, AF and CEP cells) have been shown to take on roles and markers classically expressed by immune cells (Le Maitre et al., 2005; Jones et al., 2008; Phillips et al., 2013a; Risbud and Shapiro, 2013), and thus have been described by some authors as immune responses or inflammation. However, such activity of native IVD cells is not true inflammation. Therefore, distinguishing which cases of IDD involve an immune response is important as different clinical interventions and treatments would be required.

Native disc cells produce a plethora of cytokines and chemokines which are upregulated during disc degeneration and have been shown to drive many catabolic events in the IVD (Weiler et al., 2005; Le Maitre et al., 2005; Hoyland et al., 2008; Phillips et al., 2013a; Phillips et al., 2015). A shift to catabolism is at least in part driven by the increased production of cytokines in the disc by the native cells (in an intact disc) and a combination of inflammatory cells and native disc cells following CEP and AF rupture. Phillips et al. (2015) demonstrated that NP cells express a number of cytokine and chemokine receptors and are thus able to respond in a paracrine and autocrine manner (Figure 2). Caused by different, yet not fully understood mechanisms, disc cells upregulate the expression of inflammatory cytokines such as IL-1, TNFα, IL-6, IL-8 and IL-17 amongst others creating a cytokine rich catabolic environment. IL-1 has been shown to drive the catabolic events during disc degeneration (Le Maitre et al., 2005; Weiler et al., 2005; Hoyland et al., 2008; Phillips et al., 2013a; Phillips et al., 2015). Whilst other cytokines and chemokines (e.g., MCP-1, TNFα, IL-8) produced in the disc appear to have limited effects on the native disc cells due to lack of receptor expression *in vivo* (Le Maitre et al., 2007; Phillips et al., 2015), they undoubtedly diffuse to the surrounding tissues leading to increased inflammation in local tissues, and drive cellular infiltration following AF and CEP rupture and increased sensitisation of nerves (Ye et al., 2022). Such cytokines can stimulate specific intracellular signaling pathways that further enhance the degenerative process (Daniels et al., 2017; Suyama et al., 2018) and upregulate matrix-degrading enzymes known as matrix metalloproteinases (MMPs) and a disintegrant and metalloproteinase with thrombospondin motifs (ADAMTS), specifically MMP- 1, 2, 3, 9, 13 and ADAMTS-4, 5 (Baumgartner et al., 2021c). In later phases of IDD, these cytokines can upregulate neurotrophic and angiogenic factors, which could lead to further nerve and blood vessel ingrowth (Purmessur et al., 2008; Lee et al., 2011; Binch et al., 2014; Krock et al., 2014).

Remarkably, some of these cytokines, such as IL-1, have also been shown to be expressed in cells from non-degenerate discs and display roles in maintaining normal homeostasis (Le Maitre et al., 2005; Phillips et al., 2015). Indeed, if the IL-1 agonists are knocked out during development, IDD can be induced (Gorth et al., 2019). Thus, IL-1 plays a role as a normal regulatory mechanism during IVD homeostasis, which becomes imbalanced during IDD (Le Maitre et al., 2005) (Figure 2). Native NP cells have also been shown to take on other roles normally associated with immune cells. such as phagocytosis: Jones et al. (2008) observed the capacity of bovine NP cells to ingest latex beads at least as efficiently as phagocytic cells and ingested apoptotic cells. This capability could be of great physiological relevance to maintain a healthy disc, as it may prevent inflammation triggered by the release of toxic or immunogenic intracellular content by apoptotic cells (Fadok, 1999). Clearly, the suggestion from some reviews that cytokine production within the disc is solely from immune cells is inaccurate (Ye et al., 2022). However, when the AF or CEP becomes ruptured or fissures occur during injury or disc degeneration this provides a route for blood vessel ingrowth and migration of immune cells into the intervertebral disc. Within these “non-intact” IVDs, immune cells will migrate including T cells (CD4^+^, CD8^+^), B cells, macrophages, neutrophils and mast cells (Risbud and Shapiro, 2013) (Figure 2).

**FIGURE 3:**
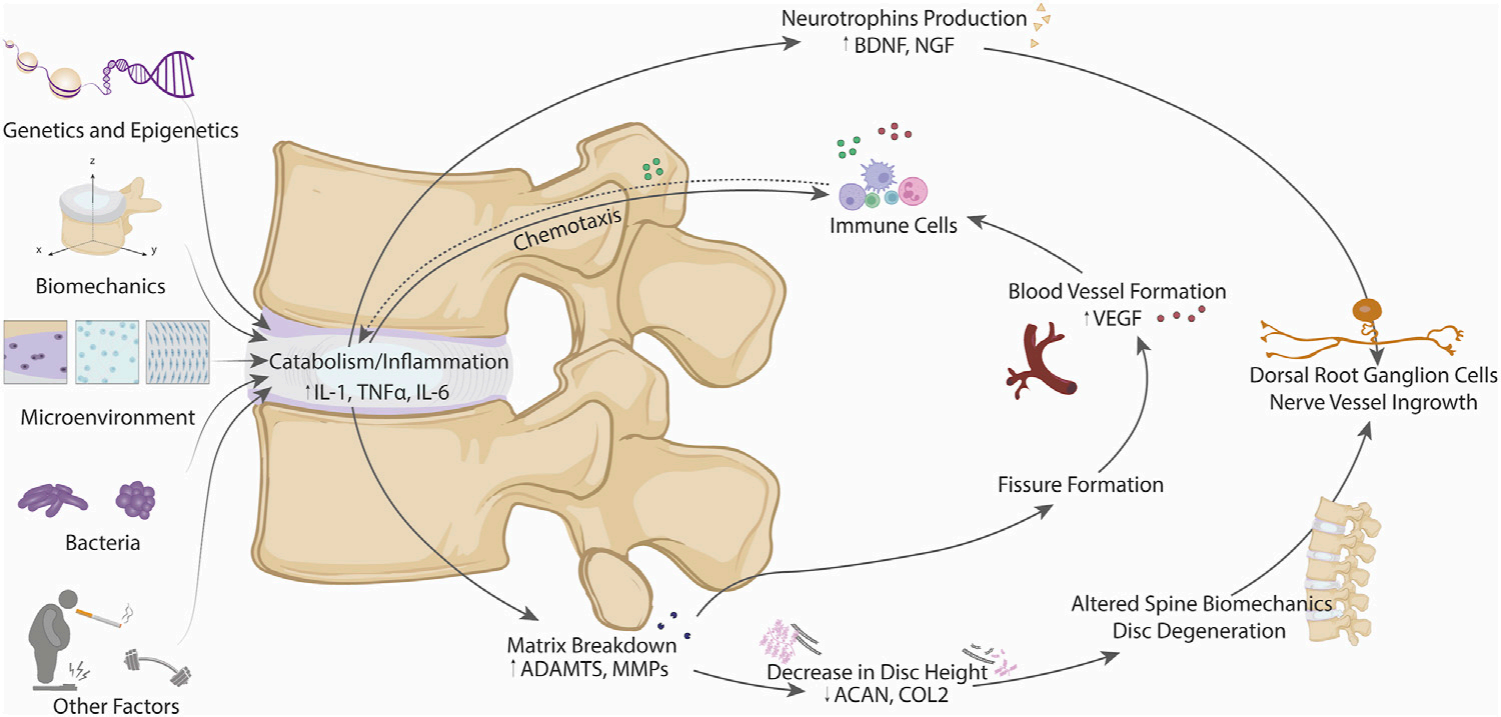
Schematic diagram of the different factors contributing to the metabolic shift from anabolism to catabolism in IDD, including genetics and epigenetics, biomechanics, microenvironment, presence of bacteria and other factors. All these contributors can promote a downstream biochemical effects (matrix breakdown and neurotrophins production) leading to structural and biomechanical alterations, nerve ingrowth and blood vessel formation. Thus, involvement of immune system could be achieved by chemotaxis losing the immuno-privileged state of the IVD.

These immune cells then contribute to an inflamed environment in the disc, leading to further increases in cytokine and chemokine expression (Phillips et al., 2015). This leads to a viscous circle of inflammatory driven catabolism which acts synergistically with the native IVD cells to cause accelerated ECM breakdown (Figure 3) (Risbud and Shapiro, 2013). These cytokines and chemokines play a number of roles within this disc, including direct actions on NP, AF and CEP cells where their receptors are present (Le Maitre et al., 2005; Le Maitre et al., 2007; Phillips et al., 2015). They will also likely diffuse out of the IVD leading to increased cellular migration to the disc (Pattappa et al., 2014), or sensitization of local nerve roots (Leung and Cahill, 2010; Johnson et al., 2015).

## The Shift From Anabolism to Catabolism

### A Complex Interplay of Microenvironment, Biomechanics, Genetics and Epigenetics, Bacterial Infection and IVD Cells

As discussed during disc degeneration, there is a shift in metabolism from anabolism (matrix synthesis) to catabolism (matrix degradation), and this shift to catabolism is accompanied by increased production of neurotrophic and angiogenic factors which lead to nerve and blood vessel ingrowth leading to inflammation and increased pain sensation in the disc (Baumgartner et al., 2021c). There remains a poor understanding as to the initiating factors involved in this switch from anabolism to catabolism in disc degeneration, which is likely due to multifactorial processes including the disc microenvironment, biomechanics, genetics and epigenetics, and even bacterial infection of the disc and the gut microbiome (Figure 3) (Li W. et al., 2022).

### Altered Disc Microenvironment

The IVD microenvironment is commonly described as harsh due to its limited nutrition (glucose and oxygen), low pH, and large changes in tissue osmolarity (Urban 2002). These factors not only impair the success of cell therapies (e.g., mesenchymal stromal cell injection) (Loibl et al., 2019; Williams et al., 2021) but can also negatively affect resident IVD cells and thus contribute to the catabolic-inflammatory shift observed during degeneration.

Limited nutrition and tissue acidity are a result of the avascular nature of the IVD (Hukins, 1988). Glucose and oxygen transport into the IVD, as well as the removal of cellular waste products such as lactic acid (which contributes to the drop in tissue pH), are hence dependent on diffusion via the capillaries in the endplate. Degeneration-associated calcification as well as a reduction in the density of the bone marrow contact channels in the endplates might further impair these transport mechanisms (Benneker et al., 2005; Chen et al., 2014). In the centre of the IVD, glucose levels, oxygen and pH can hence drop to 0.5 mM, 1% oxygen, and 6.5, respectively, although even lower values have also been reported (Urban et al., 2004). It is not clear whether glucose deprivation can activate the expression of pro-catabolic factors in NPC (Baumgartner et al., 2021b). However, multiple studies have demonstrated that glucose deprivation impairs NP cell proliferation and survival as well as proteoglycan synthesis/degradation and collagen synthesis (Bibby and Urban, 2004; Johnson et al., 2008; Chang et al., 2017; Saggese et al., 2020), and recent evidence highlights that non-coding RNAs (e.g., circ_0075062) may be involved in these processes (Chang et al., 2021). In contrast, oxygen deprivation alone was shown numerous times to have little effect on IVD cell proliferation or survival and mostly contributes to cell impairment prompted by other microenvironmental factors (Bibby and Urban, 2004; Johnson et al., 2008). Indeed, IVD cells are likely unaffected by hypoxia because of their robust and constitutive expression of hypoxia-inducible factor (HIF) 1, and more importantly, the inducible subunit HIF-1α (Merceron et al., 2014). Even more, hypoxia and HIF-1α were recently shown to attenuate the endoplasmic reticulum (ER) stress responses in NP cells (Novais et al., 2021). However, research related to rheumatoid arthritis demonstrated that the expression of Toll-like receptor 4 (TLR4) and TNF-α, but also of IL-10, are HIF-1-dependent processes in macrophages, indicating that IVD-infiltrating immune cells may be more affected by the hypoxic microenvironment than IVD cells themselves (Guo and Chen, 2020). Similar to low glucose concentrations, high lactic acid concentrations and the resulting drop in pH promotes cell death and a catabolic shift in mRNA expression (Horner and Urban, 2001; Bibby and Urban, 2004; Neidlinger-Wilke et al., 2012; Gilbert et al., 2016), likely via acid-sensing ion channels (Gilbert et al., 2016). Importantly, this response was more pronounced when cells were simultaneously exposed to low glucose levels (Bibby and Urban, 2004). Therefore, low glucose and high lactic acid levels, but not hypoxia, contribute to the catabolic shift observed during IVD degeneration. In general, there is a clear need to increase the number of experimental studies where different microenvironmental factors are combined, including both nutritional and pro-inflammatory cues (Baumgartner et al., 2021b).

Aside from low glucose, oxygen, and pH, the osmolality of the IVD is often considered a fourth harsh microenvironmental factor. This mostly refers to the relatively high osmolarity in the IVD, where 400 mOsm is considered iso-osmotic. However, it is important to note that IVD cells can be exposed to a wide range of tissue osmolarity, and these changes are more likely to affect IVD cell behaviour than the iso-osmotic condition. It can drop as low as 300 mOsm with the loss of proteoglycans during degeneration, and increase to approximately 500 mOsm during high mechanical loading (Sadowska et al., 2018). A reduction in tissue osmolarity leads to cell swelling (up to 20%) by the solubility-diffusion water transport across the cell membrane (Sadowska et al., 2018; Snuggs et al., 2021). Ample publications have shown that this hypoosmotic shift can activate and/or interplay with pro-inflammatory factors and catabolic responses and, hence, promote IVD inflammation and degeneration (Chen et al., 2002; Wuertz et al., 2007; Walter et al., 2016; Sadowska et al., 2020). Although the underlying mechanisms have not yet been identified, Transient Receptor Potential (TRP) channels and aquaporins may be involved (Sadowska et al., 2018; Sadowska et al., 2020; Snuggs et al., 2021). The hyper-osmotic shift in the IVD microenvironment leads to activation of the robustly expressed osmo-sensitive transcription factor TonEBP (tonicity-responsive enhancer binding protein) (Sadowska et al., 2018; Baumgartner et al., 2021c), which protects IVD cell viability under hyperosmotic stress (Tsai et al., 2006; Choi et al., 2018) and can also be regulated by cytokines (Johnson et al., 2017). These studies on IVD cells highlight that hyperosmolarity is likely not a main contributor to the catabolic shift in the IVD, whereas hypo-osmolarity seems to have detrimental effects on IVD cells. However, although no studies have specifically investigated the effect of osmolarity on IVD-infiltrating immune cells yet, research on other tissues (e.g., renal medulla, skin, lung epithelium) indicates that increased osmolarity activated macrophage inflammatory responses, which is at least partially TonEBP-dependent (with a threshold at approximately 360–380 mOsm) (Aramburu and López-Rodríguez, 2019). More research will hence be needed to better understand the role of the IVD microenvironment on macrophage polarization and the behaviour of other infiltrating immune cells.

### Unbalanced Biomechanics and Mechanobiology in Catabolism

Biomechanics is another key contributor in the shift from anabolism to catabolism (Adams et al., 2000). The IVD experiences various forces throughout everyday life, which are necessary to maintain the health of the disc. For instance, the average intradiscal pressure in a healthy IVD ranges from 0.1 MPa (lying prone) to 0.5 MPa (standing flexed forward) (Wilke et al., 1999). However, damage occurs in the disc when it encounters abnormal or excessive forces, leading to catabolism, including increased cytokine production, and matrix degradation (Walter et al., 2011; Vergroesen et al., 2015; Fearing et al., 2018). This damage is believed to cause microinjuries within the disc, which gradually build up over time (Adams and Roughley, 2006; Baumgartner et al., 2021c), and is likely to contribute to the infiltration of immune cells because of the chemo-attraction effect of the pro-inflammatory cytokines released by the native IVD native cells. As the tissue degenerates, the size and composition of the IVD changes, leading to impaired response to any mechanical loading placed on the disc and causing further damage, possibly leading to disc herniation or endplate defects.

As a highly hydrated tissue, the NP provides protection to compressive forces imposed on the IVD (Adams and Roughley, 2006) while the more fibrous, surrounding AF confines the NP swelling pressure and helps the IVD to resist shear and tensile forces (Chu et al., 2018). When the NP loses hydration, the compressive load is transferred to the AF (Adams et al., 1996). Whereas the healthy AF, as a whole, is highly resistant mechanically, aberrant loading can further contribute to fissure formation where the tissue is already weakened by altered turnover, which allows for associated blood vessel growth and immune cell infiltration as discussed earlier (Lama et al., 2018).

At the cell level, specific biomechanical cues have been shown to impair IVD cell response. For example, shear stress has been found to lead to increased nitric oxide, causing downstream reduction in proteoglycan synthesis and increased apoptosis in IVD cells (Liu et al., 2001). Interactions with biochemical signalling was further demonstrated. For instance, it was found that AF and NP cells from a degenerated IVD respond differently to those from a healthy disc, suggesting that mechano-transduction pathways are altered through degeneration (Le Maitre et al., 2008; Le Maitre et al., 2009a; Chu et al., 2018) and can be modulated by cytokines such as IL-1 and IL-4 (Elfervig et al., 2001a; Gilbert et al., 2011). Additionally, a pro-inflammatory environment has been shown to change the mechanobiology of IVD cells. Treatment with inflammatory stimuli, specifically liposaccharide (LPS) or TNF-α, before osmotic loading was shown to increase hydraulic permeability and cell size, disrupt the F-actin cytoskeleton, and increase aquaporin-1, which is a main water channel in NP cells (Maidhof et al., 2014). Recently, Hernandez et al. demonstrated that inhibiting actomyosin contractility in NP cells caused a similar response as TNF-α induced inflammation, while increasing contractility protected the cells against TNF-α. Actomyosin contractility was also shown to regulate nuclear factor kappa-B (NF-κB) and downstream ECM degradation, conveying that mechano-transduction and inflammatory pathways are connected and the cross-talk could play an important role in IDD (Hernandez et al., 2020b). Thus, altered biomechanics can lead to mechanobiology alterations promoting matrix degradation and impacting the capacity of the disc to sense loads normally, leading to increased catabolism and IDD development.

### Genetics and Epigenetics in IDD

Among the different causes for IDD, genetic susceptibility plays a crucial role. High heritability (over 70%) has been systematically reported for IDD (Battié and Videman, 2006; Kepler et al., 2013), as well as specific traits such as herniation (Sambrook et al., 1999) and endplate defects (Munir et al., 2018) and their progression (Williams et al., 2011b). Genetic burden, in such elevated polygenicity presented by IDD, is suggested to carry a larger effect than environmental factors, with the exception of body mass index (BMI). However, BMI itself has high heritability and polygenicity (Robinson et al., 2017), which is partially overlapped with IDD (Zhou et al., 2018).

Genetic associations for IDD have been mostly researched with candidate gene studies [see focalized reviews in Mayer et al. (2013), Feng et al. (2016), Kawaguchi (2018)]. The most representative functional group consists of genes of structural proteins and those regulating its turnover (Table 1).

**TABLE 1:** Gene variants of structural/regulatory components of IVD associated with IDD by candidate gene approach.

Structural/Regulatory component	Function	Gene	Gene variant	Molecular level	Contribution to IDD	Referernce
Collagen IX	Cartilage anabolic marker	*COL9A2*	rs7533552	-	Associated with greater disc bulging (L1-L4)	Näkki et al. (2011)
				May contribute to reduced collagen crosslinking	May contribute to disc instability and eventually prolapse in the elderly	Wrocklage et al. (2000)
		*COL9A3*	Trp3 allele in IL1B 3954 C/T variant	*COL9A3* gene on IDD might be modified by the IL 1β gene polymorphism	Influence MRI signal intensity in NP in the absence of the IL1β 3954 C/T allele	Solovieva et al. (2006)
Collagen XI	Anabolic marker	*COL11A1*	rs1676486	Lower *COL11A1* expression	High risk of herniation	Mio et al. (2007)
		*COL11A2*	rs2076311	-	Association with (i) disc signal intensity (ii) disc bulging	Videman et al. (2009)
Collagen I	AF anabolic marker	*COL1A1*	rs1800012	-	Not associated with IDD (taken as a single factor)	Anjankar et al. (2015)
				-	Risk factor related to IDD in older people	Pluijm et al. (2004)
				-	Strong association with LDD in young male	Tilkeridis et al. (2005)
Aggrecan	IVD anabolic marker	*ACAN*	*ACAN* VNTR polymorphisms	-	Increased risk of LDD of shorter alleles	Kawaguchi et al. (1999), Gu et al. (2013)
				-	Aggrecan allele with 26 repeats is associated with dark NP MRI intensity	Solovieva et al. (2007)
Cartilage Intermediate Layer Protein	Cartilage-like catabolic marker	*CILP*	rs2073711	TGF-β1 inhibition mediated induction of ECM proteins through direct interaction with TGF-β1	Association between IDD and *CILP* rs2073711 variant in women	Kelempisioti et al. (2011)
			1184T/C	-	The *CILP* SNP 1184T/C is a risk factor for male collegiate athletes	Min et al. (2010)
				-	Upregulation of *CILP* in intervertebral discs increased disc degeneration progressed	Seki et al. (2005)
Metalloproteinase	Catabolic marker	*MMP3*	Combination of the T-C haplotype of IL 1α and the MMP3 minor 5A allele	*IL1* promoted cartilage degradation through the induction of the matrix-degrading enzymes such as MMP1, MMP3, and MMP13	Association between a combination of *IL 1* and *MMP3* gene variations and type II Modic changes among middle-aged Finnish men	Karppinen et al. (2008)
			promoter 5A/6A	Enhanced the degeneration of IVD associated with environmental conditions resulting from the induction of a higher level of MMP3 expression in response to such conditions	accelerate IVD degeneration in the elderly	Takahashi et al. (2001)
			Intron 4 C/T	-	Associated with radiographic progression of LDD	Valdes et al. (2005)
		*MMP2*	1306C/T	-	Correlation with more severe grades of disc degeneration and thus may be a genetic risk factor related to LDD susceptibility in the young adult population	Dong et al. (2007)
		*MMP9*	1562 C/T	-	Associated with a high risk of degenerative disc disease in the young adult population in North China	Sun et al. (2009)
Interleukin	Catabolic marker	*IL 1β*	3954 C/T	COL9A3 gene polymorphism on IDD might be modified by the IL-1β gene polymorphism	Association between collagen gene polymorphisms and disc degeneration of the lumbar spine is modified or negatively confounded by the IL1β (C3954-T) polymorphism in middle-aged working men	Solovieva et al. (2006)
		*IL 1α*	Combination of the T-C haplotype of IL1A and the MMP3 minor 5A allele	IL1 promotedcartilage degradation through the induction of matrix-degrading enzymes such as MMP1, MMP3, and MMP13	Association between a combination of IL1 and MMP3 gene variations and type II Modic changes among middle-aged Finnish men	Karppinen et al. (2008)
			889C/T	-	IL1 gene cluster polymorphisms have an effect on the risk of disc degeneration, particularly TT genotype of the IL-1α gene promotes higher risk of disc bulges	Solovieva et al. (2004)
				Significantly increased the transcriptional activity of the IL1A gene and IL-1β protein	IL 1α −889T represented a significant risk factor for the IDD-phenotype	Virtanen et al. (2007)
		*IL 6*	rs1800797, rs1800796 and rs1800795	-	IL 6 variants are associated with moderate IDD in a sample population of young adults	Kelempisioti et al. (2011)
			597G/A, 174G/C and 15T/A	-	association analysis provided support for a link between the IL 6 sequence variants and IDD	Noponen-Hietala et al. (2005)
		*IL 18*	rs1420100	-	Association with severe degeneration	Omair et al. (2013)
Thrombospondin	ECM regulation	*THBS2*	rs9406328	lower affinity for MMP binding and thus reduces MMP degradation	Regulation of Intervertebral disc ECM metabolism by the THBS2-MMP system plays an essential role in the etiology and pathogenesis of lumbar disc herniation	Hirose et al. (2008)
A disintegrin and metalloproteinase with thrombospondin motifs (ADAMTS)	Catabolic marker	*ADAMTS5*	rs151058, rs229052, and rs162502	Decreased binding affinity with LRP1 (protein that regulates its degradation by endocytosis)	Genetic polymorphisms of ADAMTS 5 may be associated with susceptibility to LDD	Wu et al. (2014)
Growth differentiation factor 5	Pro-chondrogenic factors	*GDF5*	rs143383	-	5 population cohorts from Northern Europe indicate that a variant in the *GDF5* gene is a risk factor for LDD in women	Williams et al. (2011a)
tSKT		*KIAA1217*	11 KIAA1217 variants in Exon 2, 3, 6, 7, 13, 14, 17 and 19	-	Strong causative candidates for the Vertebral Malformation phenotypes	Al Dhaheri et al. (2020)
			rs16924573	-	Association with lumbar disc herniation	Karasugi et al. (2009)
				-	Association with lumbar disc herniation	Kelempisioti et al. (2011)
FAS receptor and ligand	Cell apoptosis factors	*FAS and FASL*	rs2234767(*FAS*) and rs763110(*FASL*)	-	FAS and FASL may be associated with the presence and severity of LDD	Zhu et al. (2011)
Caspase-9		*CASP-9*	1263A/G	-	Risk factors in the incidence of LBP in Chinese male soldiers	Mu et al. (2013)
			rs1052576	-	Associated with lumbar disc herniation and disc degeneration in the Han population of northern China	Sun et al. (2011)
Tumor necrosis factor related apoptosis-inducing ligand		*TRAIL*	1525 G/A and 1595 C/T	-	Associated with the susceptibility and severity of LDD in the Chinese Han population	Du et al. (2015)
Death receptor 4		*DR4*	rs4871857	-	Associated with the risk and severity of LDD in the Chinese Han population	Tan et al. (2012)

Among structural components, collagen variants have been extensively assessed, and several collagen types and variants have been associated with IDD. Collagen type IX polymorphisms in alpha 2 and 3 (COL9A2, COL9A3) chains have been found to influence MRI signal intensity in NP (Wrocklage et al., 2000; Solovieva et al., 2006; Zhang et al., 2008; Näkki et al., 2011). Further, Solovieva et al. (2006) stated that the effect of the Trp3 allele in COL9A3 is dependent on an IL-1B polymorphism, reflecting the effect of immune-modulators/catabolic factors on IVD degeneration. Nevertheless, the pathophysiology of this interaction is not yet described. Polymorphisms of collagen type XI (COL11) have been associated with higher risk of herniation (Mio et al., 2007; Videman et al., 2009) and other degenerative traits (Noponen-Hietala et al., 2003; Solovieva et al., 2006; Videman et al., 2009; Kalb et al., 2012). Mio et al. (2007) stated that the variant rs1676486, which falls in *cis* elements region lowers COL11A1 expression due to decreased stability of its transcripts/mRNAs.

Similarly, a polymorphism found in an intronic region of the collagen 1 gene (COL1A1) that corresponds to a binding site of Specificity protein 1 (Sp1) has been shown to increase the risk of IDD, but the mechanism is not reported yet (Pluijm et al., 2004; Tilkeridis et al., 2005; Anjankar et al., 2015). However, it has been demonstrated that Sp1 downregulates pro-inflammatory cytokine-induced catabolic gene expression in disc cells (Xu et al., 2016). Additionally, Sp1-dependent mechanisms have been reported to modulate mechanically-induced apoptosis and autophagy in IDD (Li et al., 2020). Nonetheless, Sp1 also affects processes in other tissues including differentiation, angiogenesis and chromatin remodeling (Tan and Khachigian, 2009), but its potential effects on disc cells remains yet to be identified. Interestingly, Sp1 expression is inhibited by NF-κB (Tapias et al., 2008), which has been shown to be able to initiate a pro-inflammatory cascade in other tissues, often as a reaction to extracellular stimuli (Hunter and De Plaen, 2014). And although it is possible that this dual regulation confounds the potential catabolic effect of Sp1 deprivation with an inflammatory-like response, further studies are needed to distinguish the catabolic and inflammatory responses.

Another interesting gene, Aggrecan gene (ACAN), presents tandem repeat polymorphisms in the CS1 domain. Several studies have reported that lower repeat number can lead to lower chondroitin sulfate (CS), thus linking aggrecan with IVD degeneration (Kawaguchi et al., 1999; Solovieva et al., 2007; Kim et al., 2010; Gu et al., 2013). Low CS reduces the amount of water accumulated to withstand compression loadings, reducing disc mechanical properties. Further, it is possible that lower ACAN and CS reduce the IVD’s capability to recover after acute catabolic processes (Kim et al., 2010).

Another protein, cartilage intermediate layer protein (CILP), whose expression is restricted to different cartilage(-like) tissues including IVD, inhibits TGFβ, therefore preventing the ECM anabolism and cell proliferation promoted by TGFβ in IVD (Liu et al., 2021). A polymorphism in the interaction region between CILP and TGFβ has been shown to change their binding affinity, consequently, identifying it as a risk factor for IVD degeneration (Seki et al., 2005; Min et al., 2010; Kelempisioti et al., 2011). Additionally, CILP is able to inhibit Insulin-like growth factor-1 receptor (IGFR1), acting as an antagonist of Insulin-like growth factor 1 (IGF1), a factor that mediates chondrocyte anabolism and proliferation (Liu et al., 2021). Similarly, tandem repeat polymorphisms in Asporin gene inhibits TGFβ-induced anabolism with likely synergic effects with the CILP variant (Song et al., 2008; Min et al., 2010).

As stated before, pro-inflammatory cytokines are the key factors that start the catabolic shift through increase of matrix-degrading enzymes expression, with IL-1β being one of the most influential cytokines produced by the native IVD cells and immune cells following IVD rupture (Le Maitre et al., 2005; Millward-Sadler et al., 2009; Phillips et al., 2015). As mentioned earlier, combinations of specific IL-1 β and COL9A3 polymorphisms constitute a risk factor for IVD degeneration. Similarly, a combination of MMP3 and IL-1 β polymorphisms also presents a higher risk of IVD degeneration (Karppinen et al., 2008). Additionally, other MMP-3 (Takahashi et al., 2001; Valdes et al., 2005), *MMP-2* (Dong et al., 2007) and *MMP-9* (Sun et al., 2009) polymorphisms have shown greater risk. Likewise, different single nucleotide polymorphisms (SNPs) of IL-1 β and their combinations present increased risk of IDD, possibly due to overactivation under mechanical stress (Solovieva et al., 2004; Virtanen et al., 2007; Karppinen et al., 2008). Other interleukins such as IL-6 (Noponen-Hietala et al., 2005; Kelempisioti et al., 2011) and IL-18 (Omair et al., 2013) have also been reported to increase catabolic processes in the IVD, similar to IL-1 β.

Matrix-degrading enzymes can also be regulated through their degradation. A SNP in the Thrombospondin-2 gene (THBS2), which regulates degradation of MMPs through endocytosis, has shown lower affinity for MMP binding which in turn reduces MMP degradation and increases the risk of IDD (Hirose et al., 2008). In similar fashion, different variants of diverse ADAMTS family members, which degrade aggrecan, were identified to increase risk of degeneration (Rajasekaran et al., 2014; Wu et al., 2014; Liu et al., 2016) and are increased during IVD degeneration (Pockert et al., 2009). A SNP in ADAMTS identified by Wu et al. (2014) decreases binding affinity with the protein that regulates its degradation by endocytosis, LRP1, therefore increasing its catabolic activity.

A shift toward catabolic processes can also be provoked by perturbing cell differentiation and viability. This has identified different variants of growth factors to be associated with IDD, such as Growth Differentiation Factor 5 (GDF5) (Williams et al., 2011a), which is a key regulator of matrix synthesis in the disc (Le Maitre et al., 2009b); SKT gene (KIAA1217) (Karasugi et al., 2009; Kelempisioti et al., 2011; Al Dhaheri et al., 2020), vascular endothelial growth factor (VEGF), and endothelial nitric oxide synthase (eNOS) (Han et al., 2013). Variants in factors that modulate cell apoptosis, such as FAS receptor and its ligand (FASL) (Zhu et al., 2011), Caspase-9 (Sun et al., 2011; Mu et al., 2013), tumor necrosis factor related apoptosis-inducing ligand (TRAIL) (Du et al., 2015), and Death receptor 4 (DR4) (Tan et al., 2012) have been found to be associated with IDD. However, exact mechanisms of how such variants affect cell fate and IDD are still unclear and require further investigation.

In addition to the candidate gene approaches, where the set of genes tested is preselected, a few Genome-wide Association Studies (GWAS) have been performed. GWAS is an agnostic method that tests variants covering the “whole” genome (Duncan et al., 2019). A GWAS performed by FMK Williams et al. found a variant of Parkinson protein 2, E3 ubiquitin protein ligase (PARK2) and one of Proteasome 20S Subunit Beta 9 (PSMB9) to be associated with degenerative discs (Williams et al., 2013). Those genes encode for proteins that aim to tag and degrade unwanted proteins, providing another method to remove matrix-degrading enzymes, altering the metabolic balance. Another GWAS identified a variant of Carbohydrate Sulfo-Transferase 3 (CHST3), a catabolic enzyme that catalyses proteoglycan sulfation, as a susceptibility gene for IDD (Song et al., 2013). The authors suggest that this enzyme interacts with a micro-RNA (miRNA) that targets proteins with important regulatory functions in cell-mediated immune responses, but further analysis is needed to confirm such hypothesis.

### Bacterial Infection and Disc Microbiome in IDD

Bacterial contamination has also been proposed as an important regulator of disc cell inflammation and catabolism, particularly in association to Modic changes (Gorth et al., 2015; Granville Smith et al., 2021). Despite the detection of various bacteria within isolated disc tissue, the presence of an IVD microbiome is still controversial as it has been traditionally considered as a sterile, immune privileged structure (Rajasekaran et al., 2020).

In Stirling et al. (2001), first reported the presence of anaerobic bacteria, particularly of *Cutibacterium acnes* (Gilchrist, 1900), previously known as *Propionibacterium acnes,* within the disc tissue of 43 out of 140 patients with *sciatica*. Recently, Granville Smith et al. (2021) performed a PRISMA systematic review identifying 36 articles from 34 research studies investigating bacteria in human IVDs. Bacteria were identified in 27 studies, whereas nine attributed bacterial presence to contamination. *C. acnes*, a Gram-positive anaerobic bacterium that is part of the natural skin microbiome, was the most abundant. Which is also associated with prosthetic joint infection (Jauregui et al., 2021) and was shown to be able to interact with bone cells (Aubin et al., 2017) and recently disc cells (Capoor et al., 2021). Coagulase-negative (CoNS) bacteria of the genus *Staphylococcus* Rosenbach 1884 were the second most abundant (Granville Smith et al., 2021). Inconsistencies between the identified bacteria and the prevalence of different bacteria can be partly administered to differences in tissue source (intact or herniated tissues), culture conditions (anaerobic vs. aerobic, culture time, culture media), differences in the methods used to detect bacteria, and differences in the administration of antibiotics. To date, there are few quantitative studies investigating bacterial infection to show whether bacteria are present *in vivo* or represent operative contamination.

Albert et al. (2013a) hypothesized that type 1 Modic changes in the adjacent vertebrae of herniated discs may be due to infection of the disc, highlighting the need to investigate bacteria presence in the disc. Treatment of chronic LBP and Modic changes with antibiotics has generated great controversy. It has been shown that in a certain subset of patients, antibiotic treatment was effective to reduce pain as well as disability (Albert et al., 2013a; Gilligan et al., 2021). However, this result has failed to be replicated in subsequent studies (Bråten et al., 2020).

Furthermore, the role of potential bacteria within the disc is unknown. However, previous studies have shown that LPS, a main component of Gram-negative bacteria, induces upregulation and production of various proinflammatory cytokines and matrix degrading enzymes in the NP over-activation of the NF-κB pathway (Li et al., 2016). However, most bacteria detected in the disc to date are Gram-positive and the potential influence of those bacteria on the disc remains poorly understood. Recently, Capoor et al. (2021) stimulated human IVD cells with *C. acnes* demonstrating an induction of catabolic cytokine expression by native NP cells, suggesting that at least in some individuals the increased catabolic cytokines during disc degeneration could be triggered by bacterial infection. Further work is required to understand whether bacteria are present within the disc and whether bacteria could act as a trigger to the catabolic stimuli seen during disc degeneration, and whether the gut microbiome could influence disc degeneration (Li W. et al., 2022).

### Translating Knowledge of Initiating Factors of IDD to Clinics

Understanding the roles and interactions of each of these initiating factors is essential in order to diagnose IDD early and identify suitable treatments. Current treatments alleviate pain but do not regenerate the disc, therefore regenerative strategies are urgently needed in clinics (van Uden et al., 2017).

Despite extensive research, tissue engineering strategies have had limited success in translating from preclinical models to beneficial treatments in patients as they fail to address pain (Isa et al., 2022). Precision medicine appears promising to use multiomics profiling to elucidate the pathology of IDD in each patient and prescribe an individualized treatment plan and determine which therapies could be most effective (Isa et al., 2022). However, a more comprehensive understanding of the disc microenvironment, biomechanics, genetics and epigenetics, and even bacterial infection throughout the different stages of IDD and low back pain would be necessary to evaluate which therapies or combination of therapies could be effective in a patient. In particular, there is a lack of studies on cartilage endplate regeneration therapies, despite its importance in the nutrient supply of the IVD (van Uden et al., 2017). Overall, experimental and computational models of IDD, which will be explored in the next chapter, remain critical toward developing novel treatments and regenerative therapies for IDD.

## Research Methods for Exploring the Inflammatory or Catabolic Environment of the IVD

### Experimental Models in IVD Research

**FIGURE 4:**
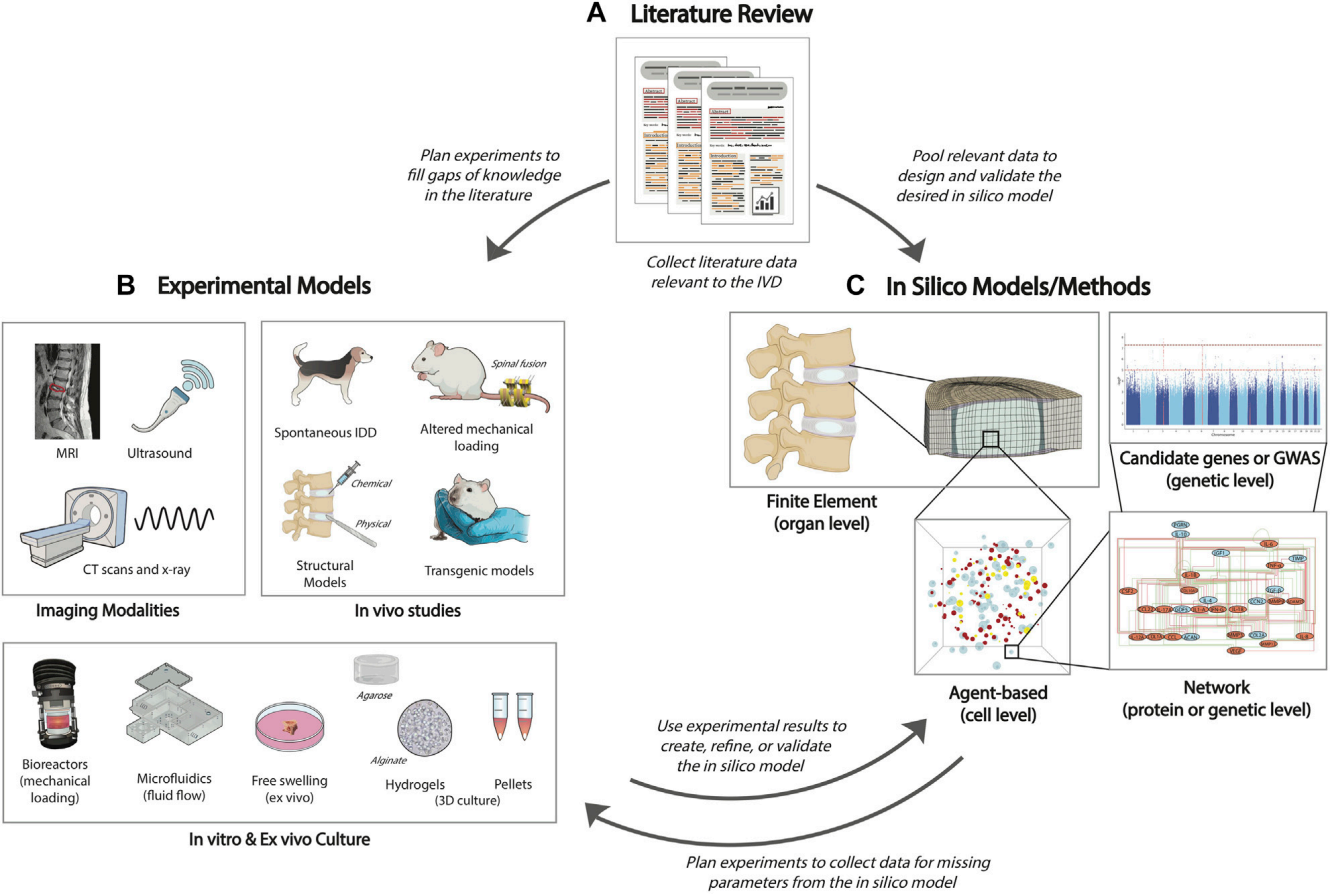
Flow chart of the intersection of experiments and computational modeling. **(A)** First, a literature review is necessary to determine the current state of the research. Then, the researcher can either perform additional experiments to fill gaps of knowledge in the literature, or use published data to create an *in-silico* model. **(B)** There are many options for experimental model design, including use of imaging modalities to view the state of the IVD, *in-vivo* animal studies which better examine the complexity of IDD, bioreactors and microfluidic devices that allow the investigation of mechanical loading or fluid flow in the IVD, and *in-vitro/ex-vivo* culture of the whole IVD or IVD cells from human or animal tissue. **(C)** *In-silico* models or methodologies can use published literature or additional experiments to provide deeper investigations into complex tissue (FEM), cell (ABM), protein (network modeling), and genetic responses (network modeling, GWAS) as well as explore interactions at multiple scales which would be difficult and expensive to do experimentally. These models can help identify novel parameters and interactions that should be validated or explored further through experiments.

For many years different experimental models have been developed for IVD research to mimic IDD. Several approaches have been used to replicate the physiological state of the IVD as closely as possible, including 3D cell and organ culture models, bioreactors and animal studies (Figure 4B). These approaches are crucial in elucidating the causes and progression of IDD, as well as in developing and testing novel therapies. Nevertheless, the best strategy to investigate IDD remains unclear, and each culture system or animal model offers different advantages and disadvantages that should be considered when planning an experiment (Table 2).

**TABLE 2:** Experimental and computational approaches applied for IDD research.

	Model	Culture system or methods	Representative studies	Scale	Parameters that can be probed	Advantages	Disadvantages	Contribution to IDD
Experimental	3D cell culture	Alginate-based hydrogels	Le Maitre et al. (2005), Hernandez et al. (2020a), Naqvi and Buckley, (2014), Le Maitre et al. (2008), Zhang et al. (2014), Wang et al. (2014), Sun et al. (2015), Öztürk et al. (2016), Thorpe et al. (2018), Lazarus et al. (2021), Rosiak et al. (2021)	Cell	Dynamic loading and catabolism induction, Stimulation with cytokines and catabolic factors	Inexpensive, non-toxic and excellent cell phenotype maintenance, human cells are available	Not recommended for AF cell culture	IVD phenotype maintenance and recovery from catabolism
		Agarose carriers	Kelly et al. (2009), Smith et al. (2011)		Dynamic loading and catabolism induction	long-term 3D culture, human cells are available	Cannot replicate macroscale forces the IVD experiences	Catabolism induction in IVD cells
		Reinforced hydrogels (Silk)	Frauchiger et al. (2017), Wöltje and Böbel, (2017)		Porosity, coating and surface area of scaffold	Biodegradable and resorbable biomaterial with high cytocompatibility	The outer layer can cause immune response	Novel therapeutic approach in IVD repair
		Pellet culture systems	Thorpe et al. (2018), Wangler et al. (2019), Hingert et al. (2019)		Hydrostatic loading and nutrient perturbation	Simple, inexpensive, human cells are available	Chondrogenic phenotype induction and lack of ECM	Effects of hydrostatic loading and nutrient deprivation in IDD
		*Ex vivo*	Bioreactors for mechanical loading	Gantenbein et al. (2006), Vergroesen et al., 2014; Costi et al., 2008, Walter et al. (2011), Paul et al. (2012), Chan et al. (2013), Paul et al. (2013), Salzer et al. (2021), Croft et al. (2021)	Organ/Tissue	Mechanical loading (compression, torsion, bending, flexion, extension, and asymmetric), environmental control (nutrition, pH, temperature, oxygen level), frequency and duration (static, dynamic, or diurnal)	Mimics physiological conditions, possibility for automation, reproducible, in line with 3R principles (“Replacement, Reduction, and refinement")	Expensive and difficult to build, culture time limited to ∼1 month, no connection to vasculature or immune system, most cannot test large sample sizes at once, human tissue is limited	Determined physiological and catabolic ranges of mechanical loading regimens and test the mechanical properties of suitable biomaterials for IVD replacement
		Perfusion bioreactors, microfluidics, “disc-on-a-chip"	Elfervig et al. (2001b), Chou et al. (2016), de Bournonville et al. (2019), Dai et al. (2019), Hwang et al. (2020), Kim et al. (2021), Mainardi et al. (2021)		Diffusion, shear stress, fluidic pattern, electrical impulses, environmental control (nutrition, pH, temperature, oxygen level)	Possibility for automation, reproducible, extends culture time, in line with 3R principles (“Replacement, Reduction, and refinement")	Cannot replicate macroscale forces the IVD experiences, difficult to design complex systems	Increased cell viability in culture provided platform to investigate cellular response to shear stress and interactions with inflammatory and neurotrophic factors, and test treatments such as electrical stimulation
		*In vivo*	Spontaneous degeneration	Daly et al. (2016)	Whole body/Organ/tissue/Protein/Genetic	Degeneration progression, therapies/treatments	Occurs naturally (therefore more ethical), immune system and pain response	Long and unpredictable time course, inherent biological and biochemical differences to humans, expensive and complex, ethical considerations	Chondrodystrophic dogs and sand rats have similar pathological changes to human in IDD and have useful in testing cellular therapies and other clinical treatments
		Altered mechanical loading	Lindblom (1957), Iatridis et al. (1999), Phillips et al. (2002), Ching et al. (2003)		Magnitude, duration, and frequency of loading	Immune system and pain response, repeatable induction of IDD at specific time point	Inherent biological and biochemical differences to humans, expensive and complex, ethical considerations	Models of bending, compression, and spinal fusion have shown that loading changes the mechanical properties of the IVD
		Structural models (physical injury or chemical injection)	Lü et al. (1997), Holm et al. (2004), Ulrich et al. (2007), Yoon et al. (2008), Dudli et al. (2011), Dudli et al. (2014), Alkhatib et al. (2014), Zhang et al. (2020), Zhou et al. (2021)		Degeneration progression, proteoglycan degradation, therapies/treatments	Immune system and pain response, repeatable induction of IDD at specific time point, useful in preclinical trials	Inherent biological and biochemical differences to humans, expensive and complex, ethical considerations, fail to capture pathogenesis of human IDD, viability of native IVD cells preserved	Critical in understanding IDD and developing/testing novel therapies for clinical application
		Transgenic models	Millecamps et al. (2011), Millecamps et al., 2012; , Li et al. (2009), Wang et al. (2012), phillips et al., 2013, Phillips et al., (2013a,b)Miyagi et al. (2014), Gorth et al. (2018)		Genes and gene pathways	Immune system and pain response, target specific pathways of interest	Inherent biological and biochemical differences to humans	Changes in SPARC, Tg197, CCN2, IL-1rn, cAct, and SMAD3 genes have been identified to contribute to IDD
Computational	Finite element		Jones and Wilcox, (2008), Newell et al. (2017), Ghezelbash et al. (2020), Galbusera et al. (2011b), Malandrino et al. (2011), Volz et al. (2022)	Organ/Tissue	Loading, environmental perturbations and catabolism induction	Valuable prediction of altered mechanics and transport at the tissue level of the IVD, 3Rs	Challenging comprehensive validation and the cellular and sub-cellular level is not contemplated. Computationally intensive and time consuming	Predictions about metabolic rates, oxygen and lactate transport, osmotic behaviour
		Agent-based	2D or 3D	Baumgartner et al. (2021a), Baumgartner et al. (2021b)	Tissue/Cellular	Cell behavior and interactions, microenvironment, time, Cell types, environmental perturbations	Dynamic, ability to model heterogenous populations, flexible, stochastic, reveals emergent phenomena	Can be computationally intensive, only as good as the rules inputted	Visual predictions of NP cells expressing TNF-α, IL-1β, or both TNF-α & IL-1β
		Network	Knowledge based or data driven	Shannon et al. (2003), Hu et al. (2004), Batagelj and Andrej, (2004), Kashtan et al. (2004), Yu et al. (2004), Schreiber and Schwöbbermeyer (2005), Wernicke and Rasche (2006), Milenković et al. (2008), Baumgartner et al. (2021a), Baumgartner et al., 2021b, Xu et al. (2021)	Protein/Genetic	Protein-protein interaction (PPI) and transcriptomic/proteomic analysis	Intuitive way to investigate, characterize, and understand interactions between biological components under micro-environmental stimuli	Difficult to construct from available IVD data which has high sample variation, different stages of IVD and type of disc tissue, and the varying methods of analysis	Capture interactions between transcriptome, proteins and their pathways in to understand the critical biochemical factors in IVD regulation. Reveal complex dynamics behind unbalanced metabolism
		Genetic analysis	Candidate gene studies, GWAS	Williams et al. (2013), Song et al. (2013), Feng et al. (2016), Kawaguchi (2018), Mayer et al. (2013)	Genetic	Genes and gene pathways	Effective in identifying genes implicated in IDD	Incapable of explaining high heritability in complex diseases due to high polygenicity and unmet “common disease, common variants” hypothesis, and due to other heritable properties as epigenetics	Identified large number of risk genomic loci involved in IDD (Table 1)
	Machine learning/AI/Deep learning	Classification of discs	Rim (2016), Niemeyer et al. (2021)	Whole body/Organ/Tissue/Protein/Genetic	Imaging, clinical categories, compound structures, gene sequence, protein/RNA data	Link seemingly unrelated entities of complex/diverse biological data	Need for algorithm creation and learning. Very subjective score system	Deep learning model for the classification of discs based on MRI with an average sensitivity of 90%
		Simplifying or coupling complex models	Pfaff et al. (2020)			Highly accurate surrogate models, significantly less computational resources and less time-consuming		

### 3D Cell Culture Systems

Over the last decades, three-dimensional (3D) cell culture models have been widely accepted due to the considerable improvements they possess in comparison to two-dimensional (2D) culture, including improved phenotypic retention to that seen *in vivo*, including: cell shape preservation; proliferation rates, and gene and protein phenotypic marker and matrix expression (Jensen and Teng, 2020). Conventional 2D monolayer culture systems lack the spatial architecture of the tissue, inducing a loss of cell phenotype and cell-ECM interactions. In contrast, 3D cultures environments promote extracellular matrix (ECM) deposition, a key factor for the maintenance of NP cell phenotype (Guerrero et al., 2020). Likewise, previous studies using notochordal cells have reported the negative effects of 2D culture (Rastogi et al., 2009) and the necessity of 3D culture system, preferably in hypoxia, and raised osmolality to maintain the phenotype (Gantenbein et al., 2014).

In the last decade many different biomaterials have been used in 3D cell culture of IVD cells, which will be listed according to the best outcome. Alginate-based hydrogels are commonly used because they are inexpensive, non-toxic and an easy 3D hydrogel model whilst maintaining excellent cell phenotype (Hernandez et al., 2020a). Notably, previous studies have reported IVD phenotype maintenance and recovery from catabolism after 3D alginate culture (Le Maitre et al., 2005; Naqvi and Buckley, 2014; Wang et al., 2014; Zhang et al., 2014; Sun et al., 2015). Additionally, alginate has been used with dynamic loading systems (Le Maitre et al., 2008) and for inducing catabolism (Le Maitre et al., 2005; Le Maitre et al., 2008). Moreover, modified alginates have shown novel properties and applications in biomedical research (Rosiak et al., 2021). For example, the sulfation of alginate hydrogel has been reported to preserve the phenotype of chondrocytes (Öztürk et al., 2016; Lazarus et al., 2021). Thus, alginate-based hydrogel systems are considered as promising biological constructs for NP cell culture (Thorpe et al., 2018). In contrast, the consistency of alginate-based hydrogels is not recommended for AF cell culture due to the lack of fibrotic structure. In addition, other materials such as agarose have been reported as long-term 3D culture models for inducing catabolism in IVD cells (Smith et al., 2011) and chondrocytes (Kelly et al., 2009). In contrast, other 3D hydrogel models such as fibrin-clots are not a realistic option for IVD 3D culture due to their lack of stiffness, despite the easy modification of this natural hydrogel. However, reinforced hydrogels with resorbable biomaterials, for instance silk, are a novel therapeutic approach in IVD repair due to high cytocompatibility (Frauchiger et al., 2017). Notably, the outer layer of silk filaments can cause immune response due to the presence of sericin (Wöltje and Böbel, 2017). 3D environment features can also be achieved without biomaterials through pellet culture. Notably, pellet culture systems have been utilized to investigate IVD degeneration, specifically examining the effects of hydrostatic loading and nutrient deprivation (Hingert et al., 2019; Wangler et al., 2019), however they induce a more chondrogenic phenotype rather than NP phenotype (Thorpe et al., 2018). Furthermore, the lack of ECM after the formation of the pellets could influence the response of hydrostatic loading (Zeiter et al., 2009), and they fail to mimic the cell-ECM connections and cell density seen in the IVD, therefore other materials would be better utilised.

In contrast, 3D constructs aim to offer a more physiological interaction between cells with ECM components without the presence of vasculature and innervation as well as the interaction with the immune system. Nevertheless, although biomaterial-based therapies have been developed in the last decades to prevent IDD, only a small number of bioengineered therapies are currently undergoing clinical trials (NCT02338271, NCT01290367, NCT01290367 and NCT02412735). Notably, pain relief does not correlate adequately with functional and structural IVD restoration. Overall, the main clinical challenge is to use clinical signs, patient pain, and disability history alongside advanced imaging techniques to design a sufficient biomaterial approach (Huang et al., 2018; Isa et al., 2022). However, *ex vivo* culture systems seem to be an appealing alternative resulting in a more representative model. Particularly, IVD explants gained attention due to a higher control of the degeneration state, sample geometry and loading (Salzer et al., 2021). Similarly, organ culture bioreactor models allow the presence of the native tissue microenvironment together with a loading system promoting a bridge between *in-vitro* and *in-vivo* models (Gantenbein et al., 2015). Such *ex vivo* culture systems are good model systems investigating intact IVDs. However, they do not enable connection with the vasculature and immune system, although co-culture systems could be developed to model these interactions.

### Bioreactors and Microfluidic Devices

Bioreactors are widely accepted as pre-clinically relevant devices that simulate the microenvironment and offer a platform to evaluate the effects of limited nutrition and incorporate more complex parameters, such as mechanical loading and fluid flow, into *in vitro and ex vivo* experiments (Haglund et al., 2011; Illien-Jünger et al., 2014; Walter et al., 2014; Gantenbein et al., 2015; Gantenbein et al., 2019; Pfannkuche et al., 2020).

Various materials or organ culture are used for different bioreactor systems. Freshly isolated IVDs from bovine tails are often used in organ culture studies due to well established operating procedures, as well as their similarities to human discs (Chan and Gantenbein-Ritter, 2012; Saravi et al., 2021). Bioreactors are also used in dynamic 3D cell culture, however the material used must be able to withstand the imposed forces. Consequently, cells are often seeded into hydrogels, such as agarose or alginate, to offer more protection against mechanical loading (Fernando et al., 2011; Cambria et al., 2020).

Initial approaches were only capable of static loading; however, over time, bioreactors have gradually evolved to become more complex, incorporating diurnal loading (Gantenbein et al., 2006) and dynamic compression (Paul et al., 2012; Paul et al., 2013; Vergroesen et al., 2014). More recently, bioreactors have advanced past compression to include two- and six-dimensional degrees-of-freedom, allowing for the analysis of torsion, bending, flexion, and extension (Costi et al., 2008; Chan et al., 2013; Croft et al., 2021). Additionally, asymmetrical complex loading has been proposed as a model to study the effects of scoliosis on disc mechanobiology (Walter et al., 2011). These increasingly complex loading devices are crucial to better understanding the effects of mechanical loading on IDD and how aberrant mechanical loading contributes to the shift to catabolism. Further, these devices are highly clinically relevant as they can test the mechanical viability of novel regenerative therapies that aim to replace or regenerate the NP, AF, and/or CEP.

In addition to mechanical loading, bioreactors are useful in simulating fluid flow to the IVD. Perfusion bioreactors have been developed to allow for *in vitro* perfusion culture of scaffold-based tissue engineering constructs, offering the ability to monitor and control key parameters such as temperature, pH, and fluidic pattern (de Bournonville et al., 2019). Microfluidic, or “organ-on-a-chip”, platforms have also been explored to study IDD and have been reviewed recently by Mainardi et al. (2021). In 2019, one of the first microfluidic “disc-on-a-chip” devices was developed by Dai et al. (2019), permitting continuous media flow to mimic the disc microenvironment, and demonstrating higher cell viability than cells in static culture, allowing for the possibility of long-term organ culture to examine chronic disc degeneration. Microfluidic devices have also been used to investigate mechanical loading in AF cells through fluid-induced shear stress (Chou et al., 2016). Studies have found that AF cells had a greater response to shear stress when stimulated with IL-1β, suggesting that disc cells are more sensitive to shear during catabolic or inflammatory conditions, possibly affecting IDD development (Elfervig et al., 2001b). More recently, electrical stimulation was tested as treatment to modulate IL-1β-mediated catabolism in NP cells (Kim et al., 2021). Additionally, a microfluidic platform was used in a co-culture system of AF, NP, and endothelial cells to investigate IDD development from inflammatory and neurotrophic factors, which could be further developed to examine pain mechanisms in IDD (Hwang et al., 2020; Mainardi et al., 2021). The ability to evaluate pain in culture systems of IVD is currently lacking, which is a major issue in translating tissue engineering strategies successfully to clinics (Isa et al., 2022). Therefore, development of a microfluidic platform that could do this would be an immense step forward toward evaluating new therapies and treatments *in vitro.* Further, microfluidic devices have been proven valuable in testing drug delivery and improving screening strategies (Damiati et al., 2018). Although these systems have been around for less time and are therefore less validated than bioreactors that offer mechanical loading, perfusion and microfluidic systems offer a promising platform to probe inflammatory and catabolic parameters and test new treatments *in vitro*.

### *In Vivo* Animal Models of Disc Degeneration

While *in vitro* and *ex vivo* models of the IVD are highly beneficial and provide insights on components of IDD, they do not offer the same level of complexity as *in vivo* studies, which may better examine the multifactorial nature of IDD and can include an immune system and pain response (Daly et al., 2016). Many animal models have been used to investigate the IVD, and the advantages and disadvantages have been reviewed extensively before (Lotz 2004; Alini et al., 2008; Showalter et al., 2012; Daly et al., 2016; Jin et al., 2018). However, no perfect model of disc degeneration currently exists, because there are many biological and biochemical differences between discs from animal species and those from humans (Oshima et al., 1993).

One major difference between human discs and animal discs is the presence of notochordal cells in the NP. In humans, notochordal cells are present at birth, but rapidly decrease and are gone by adulthood. In most other species, notochordal cells are present in adulthood. However, cows and sheep and some species of dog, classified as “chondrodystrophic”, lose their notochordal cells rapidly, similarly to humans. Although notochordal cells are not well understood, they are often considered as progenitor cells, and therefore their presence in degenerative animal models may lead to results that are minimally relevant to understanding human LDD (Alini et al., 2008). Other biochemical parameters should also be considered, such as water, GAG, and collagen content, as well as how these factors change with degeneration and age in animals versus humans, which has been reviewed previously (O'Connell et al., 2007; Beckstein et al., 2008; Miyazaki et al., 2009; Showalter et al., 2012). It should also be noted that rodents have distinctly different aggrecan proteins and do not express the same MMPs as humans, which is considered important in catabolism and tissue remodeling (Barry et al., 1994; Flannery et al., 1998). However, while rodent models may not be suitable for translational research and testing new therapies because of the major differences to humans, they offer a useful platform to elucidate the genetic basis of IDD and catabolic changes due to aging (Masuda and Lotz, 2010; Mainardi et al., 2021). Similarly, larger animal models are not a perfect match toward human IDD (Alini et al., 2008; Gullbrand et al., 2016). Nevertheless, they are furthermore suitable for initial tests of regenerative therapies as they offer valuable information on the changes in mechanical loading, whether an immune response is initiated, and possibly whether any pain is resolved.

Spontaneous degeneration occurs in mice, sand rats, chondrodystrophic dogs, and baboons; however, these models are unpredictable and often time-consuming (Daly et al., 2016). Therefore, there are various methods, categorized under mechanical or structural, that have been used to induce degeneration in animals.

In rat tails and rabbits, degeneration has been induced through altered mechanical loading, such as bending (Lindblom 1957), compression (Iatridis et al., 1999), or spinal fusion (Phillips et al., 2002; Oswald et al., 2021). In compression, the magnitude, duration, and frequency of loading cause significant changes in IVD mechanical properties, and static loading produces greater changes than cyclic loading (Ching et al., 2003).

Structural models involve a physical injury or chemical injection to the CEP, AF, or NP (Lotz 2004). Physical injuries are done using either a drill bit, scalpel, or needle. Annular injuries are commonly used and have been shown to cause decreased disc height, higher Pfirrman degeneration scores, and decreased NP GAG content (Yoon et al., 2008). Research has also shown that repetitive injury causes different inflammatory responses in the IVD. Ulrich et al. (2007) found that while a single stab injury in a rat led to localized, short-term pro-inflammatory response, while multiple stab injuries cause a prolonged upregulation of proinflammatory cytokines TNF-α, IL1-b, and IL-8 for up to 28 days after injury. CEP injuries have also been demonstrated to lead to disc degeneration similar to that of humans, characterized by decreased NP proteoglycan content and intradiscal pressure (Holm et al., 2004; Dudli et al., 2014; Zhou et al., 2021), as well as increased catabolic enzyme production and pro-inflammatory gene expression seen following CEP fracture (Dudli et al., 2011; Alkhatib et al., 2014). However injurious degeneration models fail to recapitulate the pathogenesis of human IDD and enable infiltration of inflammatory cells at a much earlier time frame than seen if at all in humans. Chemical injections with papain and chondroitinase ABC or papain are commonly used methods to induce degeneration through degrading proteoglycans in the disc (Daly et al., 2016). Although both cause catabolism, chymopapain was shown to cause greater destruction of the NP and AF proteoglycans, as well as greater spinal instability and disc space narrowing (Lü et al., 1997). However, chondroitinase ABC induced a similar catabolic shift to that seen in human IDD in the IVD of goats (Zhang et al., 2020).

In addition, groups have used transgenic animal models to represent IDD (Jin et al., 2018). The SPARC (secrete protein, acidic, rich in cysteine)-null transgenic mouse has been shown to develop behavioral signs consistent with chronic low back pain due to IDD, such as hypersensitivity to cold, axial discomfort, and motor impairment (Millecamps et al., 2011; Millecamps et al., 2012). Further, the SPARC-null mouse showed age-dependent increased innervation by sensory nerve fibers near the IVD (Miyagi et al., 2014). Gorth et al. (2018) also used Tg197 mice, a TNF-α transgenic line, to investigate the effects of systemic over-expression of TNF-α on IDD, finding that the experienced an increase in annulus tears and herniation with higher vascularity and immune cell infiltration. However, they found that intact IVDs remained healthy despite the elevated inflammation. Additionally, knockout technology has been used to create models of notochord-specific CCN2-null mice (Bedore et al., 2013), IL-1 receptor antagonist knockout mice (Phillips et al., 2013a), and β-catenin conditional activation (cAct) mice to examine the signaling pathway roles in disc degeneration (Wang et al., 2012).

Finally, emerging strategies such as tissue-engineered replacement discs have gained substantial attention in the IVD regeneration field. In terms of animal models, significant technical challenges must be addressed including cell source, construct size, culture strategies, and translational models (Gullbrand et al., 2018). Nevertheless, several studies in disc replacement, including prospective randomized comparative trials, have demonstrate advantages such as short-term superiority to spinal fusion (Hellum et al., 2012; Vital and Boissière, 2014) or at least non-inferiority to anterior spinal interbody fusion (Blumenthal et al., 2005; McAfee et al., 2005; Vital and Boissière, 2014).

### Computational Modeling of the IVD

While experimental studies are valuable in determining cell sensitivity to biochemical and mechanical cues, they are not sufficient to capture the full complexity of cell response and interactions with the microenvironment, which is crucial for understanding the transition to catabolism and the initiation of an immune response. In addition, experiments are often expensive and time-consuming. *In-silico* models can use published literature and experimental data to predict multifactorial tissue and ECM regulation at multiple scales (Figure 4). Further, *in silico* models can offer the possibility of exploring patient-specific IDD and predicting the effects and risks of available therapies prior to being treated (Rijsbergen et al., 2018). Finite element models (FEM) are useful in determining the effect of mechanical loading at the tissue and organ level, while agent-based models (ABMs) are effective in predicting tissue and cellular level changes due to the microenvironment. At the subcellular level, network modelling provides further insight into the effects of cell signaling pathways and gene variants. Machine learning and deep learning are valuable tools to analyze and classify clinical images and predict the current and future status of a patient. Each of these *in-silico* tools offers a novel way to explore catabolism or inflammation in IDD, which will be further explained in the following paragraphs.

### Finite Element Models in IDD

Finite element models (FEM) have been extensively used to represent the intervertebral disc and to simulate structural changes due to mechanical loading, providing a deeper understanding of each component’s role than what can be tested through experiments. The IVD is inhomogeneous, anisotropic, and porous, making it a highly complex structure (Newell et al., 2017). Material properties for each of IVD component, the NP, AF and CEP, are defined and validated through experimental measurements and clinical observations, however comprehensive validation of FE analysis is challenging due to the complex structure and interactions (Ghezelbash et al., 2020). The IVD components are generally modeled as a biphasic material, with an incompressible fluid phase and an elastic solid phase. Other reviews have already been written regarding FEM studies of the IVD (Jones and Wilcox, 2008; Newell et al., 2017; Ghezelbash et al., 2020), so here we will focus on how FEM has been applied to study degeneration and catabolism.

FEMs are a valuable tool to examine the vicious cycle of disc degeneration and aberrant loading. Once a disc is degenerative, the tissue biomechanics are altered, leading to a catabolic environment and causing further damage over time. However, degeneration is not uniform throughout subjects, and there are various ways previous groups have simulated degenerative discs. These include geometrical changes such as decreased height and reduced NP area (Ghezelbash et al., 2020), but also changes in the material properties, including reduced water content, calcified and thinner CEP, and a stiffer NP characterized by a decreased bulk modulus (Galbusera et al., 2011a). These studies predicted that a degenerative IVD experiences higher forces during axial rotation, as well as lower fluid flow and recovery of intradiscal pressure after loading. Models have also shown that as the NP loses fluid, it carries less load under compression, as well as with bending and shear (Ghezelbash et al., 2020). Investigation of the geometry of the IVD has concluded that simplified geometry is less stiff and does not capture the same strain distribution as FEMs based on more complex geometry obtained through segmentation of MRIs, conveying those accurate geometries are essential (Du et al., 2021).

However, there is a lack of studies that measure the effects of different patient-specific morphologies, either to observe the mechanical effects of deformation or their implications on nutrient transport. Recently, a coupled and patient-specific mechanoregulated model was developed to predict the effects of spinal fusion on disc degeneration and bone density, demonstrating how FEMs can be used by surgeons to provide insight into which patients could possibly benefit from spinal fusion treatments (Rijsbergen et al., 2018). Similarly, future models could aim to use available clinical data to help develop models that aid doctors in predicting which treatments and surgical interventions would have the best outcome.

Many FEMs of the IVD also simulate osmotic behavior, and it has been shown that a swelling model with strain-dependent osmotic pressure most accurately represents the IVD, and could be applied to investigate crack opening and fissure propagation (Galbusera et al., 2011b). A mechano-transport FEM of the IVD developed by Ruiz Wills et al. (2018) found that CEP permeability increases with aging and degeneration, and that CEP degeneration could be a cause of NP dehydration and play a key role in IDD. Other groups have simulated cell metabolism and nutrient levels in the IVD, predicting that higher cell metabolic rates lead to nutrient depletion and that application of mechanical loading led to decreased glucose levels throughout the IVD (Volz et al., 2022). Additionally, simulations of compression on oxygen and lactate transport within the IVD suggested that degenerative changes including disc height, fluid content, nucleus pressure, and cell density reductions significantly affected transport (Malandrino et al., 2011).

Overall, FEMs have proven valuable in predicting the effects of altered mechanics and transport due to degeneration at the tissue level of the IVD. However, FEMs fail to take into account what is happening at the cellular and sub-cellular level.

### Agent-Based Models

Agent-based models (ABMs) are widely used across different spatial scales and research areas. Hence, agents might reflect human beings for socioeconomic studies (Alvarez-Galvez and Suarez-Lledo, 2019) or (sub-)cellular entities in cancer research (Metzcar et al., 2019). They are particularly useful for studying complex biological processes, such as inflammation and tissue degeneration, that are dynamic, spatially heterogeneous, and stochastic. ABMs can represent individual biological cells as computational agents and can simulate how collections of cells within a tissue will respond emergently to literature-derived rules. Previously, ABMs have been shown as valuable in simulating tissue degeneration and inflammation in musculoskeletal and cardiac tissue, spanning many cell types including immune cells, fibroblasts, and stem cells (Virgilio et al., 2018; Rikard et al., 2019). Thus, ABMs offer much potential to simulate cell dynamics in cartilage tissue (Pearce et al., 2020).

In IVD research, however, ABMs have been used only recently, though initial studies have demonstrated their value in predicting IVD cell responses in a pro-inflammatory environment. Baumgartner et al. (2021c) coupled an ABM with mathematical network models (see Section “*Network Modeling*”) to investigate the relative mRNA expression of proteins and proteases in NP cells of different pro-inflammatory cell states, i.e., immunopositive for TNF-α, IL-1β, both, or none (Baumgartner et al., 2021a; Baumgartner et al., 2021b). The ABM was used to visualize cell states within the NP through predicting how immunopositive cells could be arranged within a 3D environment. Thus, it was assumed that immunopositive cells were organized in clusters based on experimental data of autocrine and paracrine stimulation (Phillips et al., 2013b; Phillips et al., 2015), short half-lives of cytokines according to distantly related studies (Kudo et al., 1990; Oliver et al., 1993; Larson et al., 2006) and diffusion of pro-inflammatory cytokines. While validation is still limited due to the lack of experimental information on the arrangement of immunopositive cells, the ABM presented a novel projection of how those cells could be spatially distributed within the NP. In this regard, ABMs can be useful in identifying novel parameters and interactions implicated in IDD and therefore guiding future experiments. Future work could extend the ABM to simulate AF and CEP cells in addition to NP cells, or to simulate advanced stages of IDD by including cell migration and adding in immune cells to further investigate the intercellular interactions in the IVD during an immune response.

### Network Modeling

Modeling biological networks provides a holistic and intuitive way to investigate, characterize, and understand the complex interactions between biological components. It is a static diagram represented by nodes (molecules) connected by lines (physical or functional interactions between nodes). The nodes are the stimuli or responses of the network, while the lines indicate either inhibition or activation between nodes directly or indirectly through other signaling pathways. The most frequently studied networks are protein-protein interaction (PPI) networks and the most commonly used software are: Cytoscape (Shannon et al., 2003), VisANT (Hu et al., 2004), TopNet (Yu et al., 2004), MAVisto (Schreiber and Schwöbbermeyer, 2005), FANMOD (Wernicke and Rasche, 2006), Pajek (Batagelj and Andrej, 2004), Mfinder (Kashtan et al., 2004), and GraphCrunch (Milenković et al., 2008).

Biological network modeling usually relies on “bottom-up” approaches, where intracellular interactions are simulated to estimate a final cellular response. Two methods can be used to build a network, either by gathering literature information regarding the pathways and the mechanisms that take part in the IVD degeneration (knowledge-based), or directly from experimental data (data-driven). In IVD degeneration, network modeling tries to capture the interactions between complex sets of proteins and their pathways and reveal the complex dynamics behind the imbalance between anabolic and catabolic processes. Identification of known NP cell high level cell regulatory factors is very important in order to integrate all the single stimuli into an IVD regulatory network model (RNM) for cell regulation, through which hypothesis and testing can be explored.

Regulatory network models can highlight the molecular signatures of the underlying pathological mechanisms that drive a condition. The reductionist view, one gene to one disease, is not applicable to a highly multifactorial condition such as IDD, thus cell signaling pathway analysis is of high importance in order to understand the system as a whole. A mechanistic understanding of the condition could pave the way for a mechanism-based biomarker selection for the effective and personalized treatment of IDD (Baumgartner et al., 2021b).

A common data-driven approach for modelling regulatory networks starts with the acquisition of -omics data from a web-based repository or by generating them. Differentially expressed genes or proteins between healthy and diseased samples are found and a functional enrichment analysis is performed in order to identify the most statistically significant pathways that are present in the condition. The final step includes experimental verification of the targets that were identified *in-silico*. However, constructing regulatory networks from IDD samples is a challenging task due to high sample variation, stage of IVD, type of disc tissue investigated and the chosen method of analysis which could be MassSpec, Microarrays or Next Generation RNA sequencing.

Xu et al. (2021) created a regulatory network behind IDD by combining transcriptomic and proteomic analysis. They hypothesized that post-transcriptional regulation could have an effect on protein content, thus, if a gene presents elevated mRNA and protein levels, it could be implicated in IDD. Their results identified six genes with these characteristics (CHI3L1, KRT19, COL6A2, DPT, TNFAIP6 and COL11A2), two of which were identified as important IDD markers in independent studies. Another group used transcriptomic data collected from lumbar-degenerated IVDs to build gene regulatory networks, finding differentially expressed genes in chemotactic signaling and matrix-degrading pathways that could later be used to help develop novel pharmacological approaches for IDD treatment (Zamanian et al., 2022). Li H. et al. (2022) constructed a protein-interaction network as well as a disease-gene interaction network that identified two potential therapeutic drugs, entrectinib and larotrectinib, demonstrating how emerging network models can be leveraged to identify novel treatments.

Recently, a top-down network modeling approach was presented to approximate cell responses of NP cells, where the cell is considered as a “black-box” (Baumgartner et al., 2021a; Baumgartner et al., 2021b). Approximations of cell responses were obtained by directly linking key relevant micro-environmental stimuli with cell responses of interest. Therefore, experimentally obtained data was systematically translated into parameters suitable for systems biology approaches. With this novel approach, interrelated results between NP cells of different pro-inflammatory states, i.e., immunopositive for TNF-α, IL-1β, or both; TNF-α and IL-1β, could be obtained for user-defined stimulus environments. This high-level network modeling methodology was embedded within an ABM (see Section “*Agent-Based Models*”) to visualize a proinflammatory environment and estimate the percentage of cells immunopositive for more than one proinflammatory cytokine, specifically TNF-α and IL-1β. Considering crucial nutritional and biochemical stimuli, *in-silico* results suggest that pro-inflammatory cytokines are important contributors in catabolic shifts in NP cell responses (Baumgartner et al., 2021b).

Top-down approaches appear promising to tackle highly complex multicellular multifactorial environments, as found in IVD tissues. Amongst others, focus might be set on the integration of more critical stimuli and cell responses in the network model.

## Methods for Genetic Analysis in IDD

In the past decades, candidate genes and Genome-wide Association Studies (GWAS) have been implemented in the discovery of genetics underpinnings of complex disorders. The former strategy involves testing the association between a particular gene variant and a trait. Therefore, the selection of the studied gene is led by *a priori* knowledge of the biological pathways that are involved in the etiology of the disease. However, the high specificity of candidate gene approaches does not reflect the polygenicity in which multiple genomic loci are involved in the development of the disease (Tabor et al., 2002).

On the other hand, the aim of GWAS is to investigate relationships between genetic variants and traits spanning the whole genome in order to give an unbiased and comprehensive view on the allelic architecture underlying complex traits (Goddard et al., 2016). Despite GWAS having identified a large number of risk genomic loci and provided valuable outcomes on the agnostic genetic discovery for complex diseases such as IDD (Uffelmann et al., 2021), there are still gaps that have to be filled. GWAS are not capable of explaining the high heritability of complex diseases. Several reasons could contribute to this limitation, such as epigenetics or epistasis, which is the phenomenon for which the effect of a genetic variant is dependent on the presence of other variants, known as genetic background. Another hypothesis is the “common variants common disease”, which considers that the genetic contribution to a disease would come from an elevated number of SNPs, each one with a very small contribution difficult to identify (Manolio et al., 2009; Gibson 2010). Moreover, the interpretation of how a specific variant affects the downstream biological pathways is very challenging. First, due to linkage disequilibrium (LD), the phenomenon for which variants close to each other are inherited together, associated SNPs are often correlated with other neighboring variants. Thus, checking LD is essential to include all potential causal variants. Further, the majority of associations discovered through GWAS (90%) fall in non-coding regions, hindering the interpretation of how such SNPs affect the phenotype (Cano-Gamez and Trynka, 2020).

For all of these reasons, when interpreting GWAS results, one should consider several factors, including the number of different associations that exist at a given locus and their LD correlation. Then it is possible to pinpoint which could be the causal variant and establish the mechanistic effects on the downstream processes (Cannon and Mohlke, 2018). Many approaches have been carried out to tackle the challenge of understanding how many signals are present at a locus, such as applying a threshold of LD.

Another approach for gathering independent variants, as conceived by Yang et al. (2012), is to perform summary-level statistics conditional analysis. Here, the effect of a lead SNP is tested for association with all the other SNPs at a *locus* to determine the degree of association between them and detect the independent signals (Schizophrenia Working Group of the Psychiatric Genomics Consortium 2014).

After different signals at a *locus* have been defined, a subsequent analysis, referred to as “fine mapping”, is performed in order to identify the potential causal variant(s). As reviewed by Schaid et al. (2018), several approaches can be adopted to perform this “fine mapping” analysis: The heuristic LD approach considers all of the SNPs that are related to the main signal with a value of LD higher than a fixed threshold. Other methods that rely more on statistics consist of jointly analyzing the correlation amongst all of the SNPs at a given *locus* through regression methods. Due to high correlation between variants, penalized regression models have been shown to be the most effective strategy. Recently, Bayesian approaches have been implemented (Jiang et al., 2019) to assess the probability of SNP causality at risk *loci* with success. When a subset of potential causal SNPs is obtained, further analyses procure the annotation of the variant biological effect, often referred as “Variant to Function” (V2F) analysis (Sun et al., 2021). So far, to relate genes to non-coding SNPs, the closest gene is considered. However, recent works (Mountjoy et al., 2021; Forgetta et al., 2022) aimed to improve this method by integrating various data sources and statistical methods to reach a better interpretation of the effect of non-coding variants on complex traits such as IDD. For this, multiple tools such as Variant effect Predictor (VEP) (McLaren et al., 2016) and Functional Mapping and Annotation of Genome-Wide Association Studies (FUMA) (Watanabe et al., 2017) can be implemented to suggest the potential molecular alterations caused by a SNP.

Additionally, an increasing number of data repositories that have collected information regarding gene expression, regulatory elements, and epigenetics, such as the Encyclopedia of DNA Elements (ENCODE) (de Souza 2012), the roadmap Epigenomics Project (Romanoski et al., 2015), and Genotype-Tissue Expression (GTEx) (The GTEX consortium, 2020) can be queried in order to obtain meaningful insights on the functional elements that could be affected by a candidate variant. In this way, one could retrieve information about the effect of the variant on the expression of a given gene. A gene-variant pair in which the variant is correlated to the gene expression is called expression quantitative trait *locus* (eQTL). Nicolae et al. (2010) demonstrated that common SNPs associated with complex diseases are significantly more likely to be eQTL, in comparison to rare SNPs acting on the phenotype by altering the expression of the gene rather than modifying the gene itself.

For instance, a study on ischemic stroke by Amini et al. (2020) showed that in blood, the SNP rs78046578 was significantly correlated to the expression of C-X-C motif chemokine ligand 10 (CXCL10), a small protein whose increased levels in the blood serum have been correlated to patients with IDD and LBP as well as with Pfirmann grades (Vazirinejad et al., 2014; Yang et al., 2022).

Moreover, Raine et al. (2014) showed that in cartilage, the polymorphism rs8031440 is correlated with the expression of SMAD3. Specifically, carrying the G allele caused a reduction in the expression of the gene. SMAD3 is a key component of the TGF-β signaling pathway, which is highly involved in the anabolism of the extracellular matrix through enhancing the expression of type I collagen (Verrecchia and Mauviel, 2004). It was reported that SMAD3 knockout mice were smaller, had malformed and kyphotic spines, and had reduced levels of collagen and proteoglycans in the disc (Li et al., 2009). Although further evidence is needed, it appears that carrying a variant that leads to a dysregulation of SMAD3 expression could lead to altered development of the disc and the spine itself.

### Machine Learning/AI/Deep Learning

Advances in computer science, computer programming, statistics, mathematics, and modelling allowed the creation of new algorithms that can “learn” and make predictions on new data. Machine learning (ML), AI, and deep learning are relatively new approaches used to tackle the complexity of a disease in order to identify biomarkers, or therapeutic interventions. The complexity and diversity of biological data (i.e., imaging, clinical categories, compound structures, gene sequence, protein/RNA data) is ideal for ML approaches to link seemingly unrelated entities.

A notable usage of deep learning in IDD is found in the classification of disc degeneration based on MRI images using the Pfirrman score. Although widely used, the Pfirrman score is very subjective and different observers sometimes classify the same image with differing scores (Rim 2016). Consistency in grading is essential for a clinician in order to have a clear idea of the patient’s condition, which has led to the development of deep learning models. Niemeyer et al. (2021) have successfully managed to develop a deep learning model for the classification of discs based on MRI data that has an average sensitivity of 90%.

Additionally, ML can be useful in studying disc degeneration through simplifying complex models to decrease computational demand, or through coupling models across several scales to offer a more holistic view of IDD. For example, while patient-specific FEMs, which were explained previously in this review, are useful in studying IVD biomechanics, they usually require complex procedures to set up and long computing times to obtain final simulation results. This therefore prevents prompt feedback to clinicians, resulting in studies with minimal sample sizes and severely hindering its suitability for time-sensitive clinical applications. As a response, neural networks have increasingly been employed in complex dynamical systems, resulting in highly accurate surrogate models that can be evaluated with significantly less computational resources and several orders of magnitude faster than conventional finite element solvers (Pfaff et al., 2020).

## Conclusion

IDD is considered a complex multifactorial and pathological disease which alters biomechanical and biochemical aspects of the IVD, resulting in a shifted metabolism associated with increased cytokine and chemokine production by native disc cells driving catabolism, this together with abnormal mechanical loading can result in disc rupture. Following rupture, immune cells are able to invade the disc, allowing crosstalk between the IVD and the immune system. Therefore, a disrupted or herniated disc is needed in order to obtain an entry point for the immune system into the disc. Nevertheless, although catabolic and inflammatory features are different, since IVD cells (NP, AF and CEP) share classical immune cell’s roles and markers (Le Maitre et al., 2005; Jones et al., 2008; Risbud and Shapiro, 2013; Phillips et al., 2013) the resulting phenotype in each case remains controversial.

In terms of terminology, immunomodulatory or inflammatory terms should only be used when immune cells are present within the IVD. Consequently, the production of chemokines and cytokines without disc rupture are contributed by the native IVD cells (NP, AF and CEPs) and should be termed catabolic cytokines and chemokines. Hence, catabolic phenotypes which are related to production of inflammatory cytokines and chemokines are incorrectly commonly attributed to inflammatory processes and easily misconstrued in the literature. Further, both processes could also simultaneously appear during IDD, making the recognition of these phenotypes more challenging.

Regarding the shift from anabolism to catabolism, it is expected that many factors are at play, including changes in the microenvironment, biomechanics, genetics, and metabolism. Within the microenvironment, low glucose and high lactic acid levels contribute to a catabolic shift in IDD, while hypo-osmolarity can activate pro-inflammatory and catabolic factors. Altered biomechanics also contributes to this catabolic shift, and aberrant loading can lead to CEP fractures and AF fissures that allow for immune cell infiltration. IDD has shown high inheritability, and gene variants in genes of structural proteins and their turnover as well as cytokines such as IL-1β have been shown to provoke a catabolic shift. While bacterial presence in the IVD is still controversial, some studies indicate that in some cases of IDD, the increased catabolic cytokines could be due to bacterial infection and treatment with antibiotics could be effective to reduce pain although the results are varied. Overall, IDD is highly multifactorial, and each of these factors discussed play a role in the shift to catabolism within an intact disc and possible immune cell infiltration following AF or CEP rupture.

However, for the best clinical treatment, early diagnosis is crucial, which means that data analysis must be streamlined and the disc pathology must be classified correctly. Therefore, it is necessary to understand how different methodologies have been used to study the different features of IDD and assign the correct terminology. For that purpose, a wide range of *in-silico, in-vivo* and *in-vitro* models have been discussed in this review to select the best approaches for future IDD studies and thus provide the best clinical output. While 3D cell culture is effective in investigating individual parameters within IDD, bioreactors and microfluidics studies offer another level of complexity through the addition of mechanics and/or fluid flow. Further, animal models provide even more sophistication as they include interactions between the disc and other tissues, however there are still many biochemical and biological differences in comparison to IDD in humans. This is in part how *in-silico* studies can be useful, as they can predict changes in the IVD based on prior research, without harming humans. Additionally, computational modeling can offer insights into IDD that are difficult or expensive to obtain through experiments. FEMs are useful to determine the biomechanical effects on the disc, which are expected to simulate specific patient models and observe the effects of CEP shape and its implications on nutrient and water transport, as well as on the different NP morphologies. ABMs can offer visual and spatial predictions, and network models provide insight into complex interactions at the protein or genetic level. Moreover, methodologies using candidate genes and GWAS have identified influential gene variants in IDD. Machine learning can then be a useful tool to simplify these models and methodologies, or to streamline and reduce the bias in the classification of IDD.

Hence, this review summarizes the recent advances of cross-disciplinary approaches to identify the mechanisms of the shift of anabolism to catabolism in the progress of IDD and compared them with immunomodulatory features. It demonstrates our current knowledge of the interplay of the immune system, metabolism, genetics, epigenetics, physiology, and mechanics, as well as computational and experimental models used to investigate catabolism and inflammation in the IVD.

## Citation

Bermudez-Lekerika P, Crump KB, Tseranidou S, Nüesch A, Kanelis E, Alminnawi A, Baumgartner L, Muñoz-Moya E, Compte R, Gualdi F, Alexopoulos LG, Geris L, Wuertz-Kozak K, Le Maitre CL, Noailly J and Gantenbein B (2022) Immuno-Modulatory Effects of Intervertebral Disc Cells. Front. Cell Dev. Biol. 10:924692. doi: 10.3389/fcell.2022.924692

## Glossary

ABM: Agent-based model
ADAMTS: a disintegrant and metalloproteinase with thrombospondin motifs
AF: Annulus fibrosus
AI: Artificial intelligence
BNB: Blood NP barrier
BMI: Body mass index
CEP: Cartilage endplate
CLIP: Cartilage Intermediate layer protein
CoNS: Coagulase-negative Staphylococci
CS: Chondroitin sulfate
CXCL10: C-X-C motif chemokine ligand 10
DR: Death receptor
ECM: Extracellular Matrix
ENCODE: Encyclopedia of DNA Elements
ENOS: Endothelial nitric oxide synthase
eQTL: expression quantitative trait *locus*
ER: Endoplasmic Reticulum
FEM: Finite element model
FUMA: Functional Mapping and Annotation of Genome-Wide Association Studies
GAG: Glycosaminoglycan
GCN: Graph convolutional networks
GDF: Growth differentiation factor
GTEx: Genotype-Tissue Expression
GWAS: Genome-wide Association Studies
HIF: Hypoxia-inducible factor
IDD: Intervertebral disc degeneration
IGF: Insulin growth factor
IGFR: Insulin growth factor receptor
IL: Interleukin
IVD: Intervertebral disc
LBP: Low Back Pain
LD: Linkage disequilibrium
LDD: Lumbar Disc Degeneration
LPS: Lipopolysaccharide
miRNA: micro-RNA
MC: Modic change
ML: Machine learning
MMP: matrix metalloproteinase
MRI: Magnetic resonance imaging
NFκ-B: nuclear factor kappa-B
NP: Nucleus pulposus
SNP: Single nucleotide polymorphism
SP1: Specificity Protein 1 (syn. Sp1 transcription factor)
SPARC: Secrete protein, acidic, rich in cysteine
THBS2: Thrombospondin-2 gene
TonEBP: Tonicity-responsive enhancer binding protein
TGF: Tumor growth factor
TNF-α: Tumor necrosis factor alpha
TRAIL: TNF-α-related apoptosis-inducing ligand
TRP: Transient receptor potential
V2F: Variant to Function
VEP: Variant effect Predictor
VEGF: Vascular endothelial growth factor
